# Opportunities for Earth Observation to Inform Risk Management for Ocean Tipping Points

**DOI:** 10.1007/s10712-024-09859-3

**Published:** 2024-11-06

**Authors:** Richard A. Wood, Jonathan A. Baker, Grégory Beaugrand, Jacqueline Boutin, Alessandra Conversi, Reik V. Donner, Ivy Frenger, Eric Goberville, Hakase Hayashida, Wolfgang Koeve, Karin Kvale, Angela Landolfi, Wieslaw Maslowski, Andreas Oschlies, Anastasia Romanou, Christopher J. Somes, Thomas F. Stocker, Didier Swingedouw

**Affiliations:** 1 Met Office Hadley Centre, Exeter, EX1 3PB UK; 2 Laboratoire d’Océanologie Et de Géosciences UMR 8187, LOG, CNRS, University of Lille, University of Littoral Côte d’Opale, 62930 Wimereux, France; 3 Sorbonne Université, CNRS, IRD, MNHN, Laboratoire d’Océanographie et du Climat: Expérimentations et Approches Numériques, LOCEAN/IPSL, Paris, France F-75005; 4 National Research Council of Italy, CNR–ISMAR, Forte Santa Teresa, Loc. Pozzuolo, 19032 Lerici, SP Italy; 5 Magdeburg-Stendal University of Applied Sciences, 39114 Magdeburg, Germany; 6 Potsdam Institute for Climate Impact Research (PIK)–Member of the Leibniz Association, 14473 Potsdam, Germany; 7 GEOMAR Helmholtz Centre for Ocean Research Kiel, 24148 Kiel, Germany; 8 Unité Biologie Des Organismes Et Ecosystèmes Aquatiques (BOREA), Muséu Unité Biologie des Organismes et Ecosystèmes Aquatiques (BOREA), Muséum National d’Histoire Naturelle, CNRS, IRD, Sorbonne Université, Université de Caen Normandie, Université des Antilles, Sorbonne Université, 75006 Paris, France; 9 Application Laboratory, Japan Agency for Marine-Earth Science and Technology, Yokohama, 236-0001 Japan; 10 GNS Science, 1 Fairway Ave, Lower Hutt, 5013 New Zealand; 11 National Research Council of Italy, CNR–ISMAR, 00133 Rome, Italy; 12 Naval Postgraduate School, Monterey, CA 93943 USA; 13 Dept of Applied Physics and Applied Mathematics, NASA Goddard Institute for Space Studies, Columbia University, New York, NY 10027 USA; 14 Climate and Environmental Physics and Oeschger Centre for Climate Change Research, University of Bern, 3012 Bern, Switzerland; 15 EPOC, UMR 5805, Bordeaux INP, University of Bordeaux, CNRS, 33600 Pessac, France

**Keywords:** Earth observation, Ocean circulation, Ocean deoxygenation, Ocean ecosystems, Tipping point

## Abstract

As climate change continues, the likelihood of passing critical thresholds or tipping points increases. Hence, there is a need to advance the science for detecting such thresholds. In this paper, we assess the needs and opportunities for Earth Observation (EO, here understood to refer to satellite observations) to inform society in responding to the risks associated with ten potential large-scale ocean tipping elements: Atlantic Meridional Overturning Circulation; Atlantic Subpolar Gyre; Beaufort Gyre; Arctic halocline; Kuroshio Large Meander; deoxygenation; phytoplankton; zooplankton; higher level ecosystems (including fisheries); and marine biodiversity. We review current scientific understanding and identify specific EO and related modelling needs for each of these tipping elements. We draw out some generic points that apply across several of the elements. These common points include the importance of maintaining long-term, consistent time series; the need to combine EO data consistently with in situ data types (including subsurface), for example through data assimilation; and the need to reduce or work with current mismatches in resolution (in both directions) between climate models and EO datasets. Our analysis shows that developing EO, modelling and prediction systems together, with understanding of the strengths and limitations of each, provides many promising paths towards monitoring and early warning systems for tipping, and towards the development of the next generation of climate models.

**Article Highlights**

- We review scientific understanding of ten potential tipping points in ocean circulation, biogeochemistry, and ecosystems
- We identify opportunities for Earth Observation to support improved models, predictions and early warning of these tipping points
- We identify how EO data can be combined with in situ data and models to maximise the value of each

## Introduction

The possibility of tipping behaviour in the ocean has a long history, heading back at least to the pioneering work of Stommel (1961), who identified the potential for bistability and threshold behaviour in an idealised two-box ocean circulation model that is controlled by density differences arising from temperature and salinity variations. At the time, Stommel did not have a specific application in mind, although he noted the important role of salinity in the circulations of the Mediterranean and Red Seas. Only much later did oceanographers realise the potential applicability of these ideas to global-scale flows such as the Atlantic Meridional Overturning Circulation (AMOC), and hypothesise their role in major palaeoclimatic variations (Rooth 1982; Broecker et al. 1985; Broecker 1987, see also Stocker et al. 2024). In more recent times, the potential of tipping behaviour or regime shift has been recognised in a much wider range of elements of the ocean-climate system, including more regional circulation elements, elements of ocean biogeochemical cycles, and ocean ecosystems (e.g. Swingedouw 2020).

While a number of definitions of ‘tipping elements’ (TEs) and ‘tipping points’ (TPs) have been used in the literature, we consciously adopt a rather loose definition of a TE here as an element of the Earth System whose response to a smoothly varying change in forcing includes an abrupt, discontinuous or irreversible change when the forcing passes a certain threshold (the TP). This definition brings together a number of responses that are similar at the coarse level, but it will remain important when discussing each individual phenomenon in depth to characterise the tipping behaviour more precisely. Some key questions for each TE include: what is the relevant control parameter/forcing?; is there a critical threshold in the forcing (bifurcation) or simply a nonlinear response to forcing?; if a threshold is crossed, is there an element of irreversibility/hysteresis if the forcing is returned below the critical value?; what is the timescale for adjustment to the new state if a threshold is crossed?; if the forcing is reduced back to a subcritical value quickly enough, could tipping be avoided (resilience time)?; could internal ‘noise’ or particularly rapid forcing changes make transition to a new state more likely?

Many of the TPs discussed here can be considered examples of ‘high impact, low likelihood’ (HILL) climate hazards. In many cases uncertainty in current scientific understanding is so deep that it is not possible to provide robust estimates of how likely the events are to occur. Such hazards pose particular risks to society because they may imply changes that are greater than society’s planned capacity to adapt to climate change, in some cases being even in the opposite direction to the expected changes. Adaptation measures may be extremely costly or have undesirable side effects, and investing in such measures when they probably will not be needed is not attractive. Despite these uncertainties, it may be possible to develop tools that allow management of such HILL risks, such as storylines of the impacts that would occur if the TP were crossed, and observable early warning indicators (EWIs) that give advance notice that the TP is being approached (Wood et al. 2023). The availability of reliable early warning of such events would enable greater flexibility in responding to the risks, while avoiding the costs of building resilience to an event that might never occur (see e.g. Marchau et al. 2019). Earth Observation (EO, taken here to refer to satellite observations) has the potential to provide important elements of these tools, including direct monitoring of the TE itself, monitoring of EWIs, and improved understanding of the processes and mechanisms at play in the TE, that can be used to constrain and refine models.

To derive the full value from EO, it is frequently important to combine the EO data with in situ observations and/or models. This can be done through: multivariate analysis of observations (which typically requires some form of simple underlying process-based model); assimilation of multiple observable types into more complex models such as General Circulation Models (GCMs), to provide either a self-consistent estimate of past variations (reanalysis), or an initial state to initialise forecasts/hindcasts of the ocean state; or confrontation of free-running GCMs (no data assimilated) with the observations, in order to evaluate the models themselves and point to paths for improvement. Assimilation of multiple data types is challenging as the error and covariance characteristics of the different variables need to be consistently specified. In this paper we aim to focus on EO variables, while addressing the developments needed in modelling, data assimilation and in situ datasets to deliver the full scientific potential.

This paper arises from discussion among a subset of the authors at the International Space Science Institute (ISSI) Workshop on “Tipping Points and Understanding EO data needs for a Tipping Element Model Intercomparison Project” in October 2022. We present a perspective of ocean TPs, focussing on opportunities to exploit existing EO data or potential new missions, to: monitor ocean tipping elements; provide useful early warning (EW) of tipping; and evaluate and improve models. The paper builds on the previous study of Swingedouw et al., (2020), by considering more (ocean) TEs, and more potential EO applications (not just early warning). We consider ten potential TEs (see below). The selection of TEs reflects to some extent the expertise of the author team, but there is a focus on TEs that are either intrinsically large-scale, or where more localised TEs are expected to be relevant over a wide geographical range. We are nevertheless conscious that some other potential ocean TEs or abrupt changes have been noted in the literature, e.g. acidification and carbon cycle (Heinze et al. 2021; Heinze et al. 2023), Mediterranean convection sites (Schneider et al. 2014), North Sea circulation (Holt et al. 2018), occurrences of marine heat waves (Benedetti-Cecchi 2021), tropical reefs (Lenton et al. 2019). We also do not discuss here impacts thresholds in coastal systems such as mangroves, sea grasses and coral reefs.

In Sect. 2, we consider six potential tipping elements of large (at least basin) scale ocean circulation and biogeochemistry: Atlantic Meridional Overturning Circulation (SubSect. 2.1); Atlantic Subpolar Gyre convection and circulation (2.2); Beaufort Gyre fresh water storage (2.3); Arctic halocline (2.4); Kuroshio large meanders (2.5); and large-scale deoxygenation (2.6). Section 3 discusses four additional potential tipping elements in pelagic ecosystems that are themselves more local/regional, but where processes may be applicable to multiple geographical regions: phytoplankton (3.1); zooplankton (3.2); higher trophic levels and fisheries (3.3) and overall biodiversity (3.4). For each of the ten tipping elements considered, we provide a perspective on current knowledge of the nature of the tipping element (processes, timescales, drivers and impacts of tipping), and needs and opportunities for EO to advance knowledge of and resilience to the risks of tipping. In Sect. 4 we synthesise the results of the previous sections, and suggest possible ways forward for the use of EO data in this field.

## Tipping Points of Large-Scale Ocean Circulation and Biogeochemistry

### Atlantic Meridional Overturning Circulation (AMOC)

#### Nature of Tipping Point

The Atlantic Meridional Overturning Circulation (AMOC) is a global-scale system of currents which transports warm water northwards in the Atlantic Basin. Much of the heat is lost to the atmosphere in the North Atlantic high latitudes, and consequently the AMOC has a fundamental role in shaping Northern Hemisphere climate (e.g. Bonnet et al. 2021). The AMOC is often characterised as a global-scale ‘conveyor belt’ circulation (Broecker 1991), driven by heat and fresh water exchanges with the atmosphere, and the consequent density changes. While this view is undoubtedly a gross simplification, model studies suggest that on decadal and longer timescales, coherent basin- or global-scale variations in the circulation are possible. In this section, we focus on large-scale variations whereas North Atlantic subpolar gyre variability is discussed in Sect. 2.2.

The AMOC has been long recognised as one of the major tipping elements of the climate system (Stommel 1961; Rooth 1982; Rahmstorf 1996, Lenton et al 2008 Swingedouw 2020; Armstrong McKay et al. 2022; Boers et al 2022). AMOC transitions (tipping) and multiple stable states have been found in a hierarchy of models, ranging from simple box models through to comprehensive General Circulation Models (GCMs). However not all climate models exhibit bistability (e.g. Schiller et al. 1997), highlighting considerable variation among climate models which persists to the current (CMIP6) generation (Jackson et al 2023). See Weijer et al. (2019) for a review.

Abrupt or gradual shifts in AMOC strength have been also identified in the paleoclimate record. For example, AMOC bi-stability has been proposed as a mechanism for rapid climate variability between glacial and interglacial periods in the Pleistocene (Dansgaard et al. 1993; Blunier et al 1998; Renssen et al. 2001; de Abreu et al. 2003; Lynch-Stieglitz 2017; Moffa-Sanchez et al. 2019), and during the last interglacial (Galaasen et al. 2014). The Younger Dryas cold event has been associated with AMOC collapse following the discharge of glacial lakes (Broecker et al 1985, 1987; Rooth 1982; Lehman and Keigwin 1992; O’ Hare et al. 2011; Rind et al 2018). AMOC variations are also thought to drive the “bipolar seesaw” leading to changes of opposite phase in the Arctic and Antarctic climates (Broecker 1998; Stocker 1998; Rahmstorf 2002; Stocker and Johnsen 2003, Skinner and Elderfield 2007; Pedro et al. 2011).

The wide range of model behaviour, and plausibility of abrupt AMOC changes, leads to a pressing need for observations to constrain the models, and to provide independent warning if such an event is approaching.

##### Drivers of AMOC Tipping

The main mechanism that drives abrupt AMOC changes in models is the salt advection feedback: if the overturning circulation carries relatively salty waters into the North Atlantic, an AMOC slowdown would tend to make the Atlantic basin fresher, amplifying the slowdown itself. Early comprehensive climate models were judged to have a ‘too-stable’ AMOC due to biases in the fresh water transport by the AMOC (e.g. Liu et al. 2017). These biases are still present in more recent climate models, although advances in modelling and resolution have reduced them to some extent (Deshayes et al. 2013; Mecking et al. 2016; Hirschi et al 2020).

However overall AMOC stability depends on the combined effect of several positive and negative feedbacks (e.g. Vellinga and Wood 2002; Yin et al. 2006; Swingedouw et al. 2007; Hofmann and Rahmstorf 2009; Hirschi et al. 2020). The AMOC only tips when the destabilising effect of the salt advection feedback overcomes the stabilising feedbacks (Jackson et al. 2017).

Key additional processes include the freshwater transport by the wind‐driven gyre circulation in the South Atlantic (Thomas and Fedorov 2019) and North Atlantic (Wood et al. 2019), discharges of excess freshwater through Fram or Davis straits, triggered by changing winds (Haine et al 2015), cooling over the Atlantic Sub-polar Gyre (SPG) due to storm track changes (Li et al 2022a), North Atlantic Oscillation (NAO) variability (Klus et al 2018), sea-ice interactions (Swingedouw et al. 2007), the strength of the ‘warming hole’ (Keil et al 2020), the rate of freshwater input (Wood et al 2019; Kim et al 2021) and other atmosphere–ocean feedbacks which may be subdued, underestimated or biased in many models (Gent 2018). Some such processes (e.g. fresh water input from the Greenland Ice Sheet) are still only incompletely represented in current climate models (Swingedouw et al. 2007; Bakker et al. 2016).

The location and likelihood of tipping in climate models depends on model parameters that are poorly known and need to be constrained using parametric uncertainty analysis. Simpler models can help here, provided they can be shown to capture the key AMOC tipping processes and feedbacks. Parameters can be estimated either by formulating the simpler models in terms of observable quantities (Wood et al. 2019), or by Bayesian ensemble inversion methods (e.g. Lux et al (2022), which showed that suitable paleo-proxy timeseries may contain enough information to constrain the likely position of AMOC TPs). By identifying the crucial parameters to constrain the position of TPs, it may be possible to focus observational efforts to provide early warning. But these problems are still very much at the research stage.

It is not clear whether there is a critical level of global warming that would trigger AMOC collapse. Based on a literature survey, Armstrong McKay et al (2022) estimate a very broad range of 1.4 to 8 °C for such a threshold, if it exists. Other factors not strongly correlated with warming level (e.g. response of the hydrological cycle to warming) may play a key role, partly explaining the wide range of warming levels suggested by Armstrong McKay et al. The results of Romanou et al (2023) suggest that even moderate climate forcing (such as SSP2-4.5) may be sufficient to drive the AMOC over a threshold.

Noise-induced transitions, in which internal climate variability causes a temporary AMOC collapse, have recently been shown to be possible in future climate simulations (Castellana and Dijkstra 2020). Romanou et al. (2023) in a fully coupled climate model and without applying external freshwater forcing, found evidence of such noise-induced transitions in a state-of-the-art climate model (NASA-GISS-E2-1-G), spontaneously arising from stochastic variability of high latitude freshwater fluxes into the ocean and with an AMOC off state lasting for about 800 years. Observational requirements to initialise prediction of such behaviour are not currently known.

Rate-induced tipping (Stocker and Schmittner 1997; Alkhayuon et al. 2019; Ritchie et al. 2022) might also occur: too-fast forcing variations (e.g. greenhouse gases or fresh water input) may cause the AMOC to tip, even before the forcing reaches a critical level. This implies that safe climate mitigation pathways need to consider rates of change as well as the final destination. Further, given that future mitigation strategies may eventually lead to drastic reductions to GHGs, more understanding is needed of potential abrupt changes under ‘overshoot’ or negative emissions scenarios (e.g. Schwinger et al 2022).

##### Timescales of Tipping

Based on a literature survey, Armstrong McKay et al. (2022) suggest a timescale for AMOC collapse, once a threshold is passed, of 15–300 years, consistent with timescales estimated from a range of model studies (Bryan 1986; Jackson and Wood 2017; Weijer et al. 2019; Sinet et al. 2022).

As discussed earlier, models might be too stable (Liu et al. 2017), and the processes that spread the freshwater discharge from GIS might be too slow (Swingedouw et al. 2021), hence the timescales from CMIP6 (Coupled Model Intercomparison Project, Phase 6) models are possibly too long. Shorter timescales were inferred from the paleoclimate record; for example the termination of the Younger Dryas cold event took only 10 years (Alley and Agustdottir 2005). The Intergovernmental Panel on Climate Change’s (IPCC’s) 6th Assessment Report (IPCC 2021, referred to as AR6) concludes that the AMOC will not collapse within this century, but only assigns ‘medium confidence’ to that conclusion, highlighting the poor capabilities of present-day models to represent a number of crucial processes.

##### Impacts of Tipping

A shut-down of the AMOC would have global impacts on climate. As well as the well-known impacts over the Euro-Atlantic sector (e.g. Jackson et al. 2015; Bellomo et al. 2023), it would lead to more extensive Arctic ice and warming of the Southern Hemisphere. The Inter-tropical Convergence Zone (ITCZ) would be expected to migrate southward and the global water cycle perturbed with weaker Indian and Asian summer monsoons (Buckley and Marshall 2016; Wassenburg et al 2021). El Nino-Southern Oscillation (ENSO) variability may be reduced (Orihuela-Pinto 2022) and shifted eastwards (Williamson et al 2018). Sea levels would rise on the western side of the North Atlantic and Atlantic storm tracks shift northward and become more intense (IPCC 2019a, b).

These effects would be expected to lead to a decrease in marine productivity in the North Atlantic, a reduction in Sahelian, South Asian and part of Amazonian rainfall, and a decrease in the number of tropical cyclones in the Atlantic (IPCC 2019a, b). While detailed sectoral impact studies are few (e.g. Ritchie et al. 2020), the societal impacts would be expected to be far-reaching (e.g. DeFrance et al. 2017).

##### AMOC Early Warning Signals and Proxies

As discussed in Swingedouw et al. (2020), to monitor AMOC shifts and identify possible TPs we need adequately long records, e.g. of temperature and salinity in the North Atlantic and transports across various latitudes (as in Frajka-Williams et al. 2019). However that may still not be sufficient to obtain early warning signals (EWS). Since direct AMOC observations have only become available fairly recently, it is likely that practical early warning signals will need to use various proxies or fingerprints of the AMOC to obtain long enough samples.

Low order dynamical system theory suggests that near a bifurcation point systems become more sluggish in response to small perturbations (Held and Kleinen 2004; Carpenter and Brock 2006; Boulton et al 2014) i.e. increasing autocorrelation and variance. A few studies have highlighted that recent changes in various AMOC proxies/fingerprints, might be exhibiting such a change in autocorrelation properties (Boers 2021; Michel et al. 2022), highlighting the potential approach of the AMOC to a tipping point. Because a long enough historical time series of the AMOC is not available, these studies unavoidably depend on proxies or ‘fingerprints’, i.e. spatio-temporal observations of other variables that are correlated with AMOC changes, e.g. SST (Dima and Lohmann (2010), Rahmstorf et al. (2015), and Caesar et al. (2018)), subsurface hydrography (Chen and Tung 2018; Fraser and Cunningham 2021), EO gravity data (Landerer et al. 2015), and surface altimetry in combination with various in situ observations (Willis 2010; Frajka-Williams 2015; Mercier et al. 2015). Both the consistency of the proxies (e.g. Little et al. 2020; Kilbourne et al. 2022) and their relevance for AMOC tipping as opposed to recent variability (Jackson and Wood 2020) remain the subject of debate. Meanwhile more process-based early warning indicators (e.g. Alkhayuon et al. 2019) are at an early stage of research.

#### EO needs and opportunities

EO can play a valuable role in monitoring the AMOC and providing insights into key processes. However, at least on the 5–10 year time horizon, it will be essential to use EO alongside key in situ datasets, (trans-basin arrays, Argo, Arctic Ocean exchanges), which observe sub-surface signatures of the AMOC that are inaccessible to satellite instruments.

Long-term consistency and continuity of existing datasets is particularly critical because the (at least) decadal timescale of AMOC tipping means that early warning indicators could be contaminated by data inhomogeneities.

##### Proxies

Proxy indicators (‘fingerprints’) of the AMOC give the opportunity to monitor in future at locations where trans-basin sections are not available or practical, and potentially allow longer past timeseries to be constructed, opening up greater options for early warning. SST, SSH and gravity observations have been identified as particular opportunities for EO. However there is substantial disagreement between different proxy timeseries, while modelling (Jackson and Wood 2020) suggests that no single proxy can capture all aspects of AMOC variability (natural variations, response to global warming, tipping).

- Work is therefore needed to develop *robust, multi-variate* AMOC proxies, probably combining EO and in situ data, for monitoring and attribution of future AMOC change.
- Machine learning, trained on a range of model data, potentially provides novel opportunities to develop such multivariate proxies (e.g. DelSole and Nedza 2022; Solodoch et al. 2023; Michel et al. 2023).

##### Processes

Improved estimates of heat and fresh water exchanges with the subpolar basin will allow improved monitoring of the drivers of AMOC change and hence potential early warning of major changes. This includes surface heat and freshwater fluxes, water inputs from rivers and the Greenland ice sheet, and Arctic exchanges.

- Key regions for monitoring include freshwater and ice transports across Fram/Denmark and Davis Straits as well as storage of freshwater in the different subpolar basins (Haine et al 2015) and ice edge/melting rates, in order to estimate changes in freshwater budgets of the deep water formation sites (Lobelle et al 2022). The potential for EO to inform ice transports in particular is discussed in Sect. 2.3.2.
- Polar regions, western boundary currents, storm tracks, and the subtropical-subpolar transition zone in the North Atlantic (Buckley and Marshall 2016 and references therein) are key regions for surface heat and freshwater exchanges. Advances may best be delivered through intensive, localised multi-platform campaigns, combining EO with in situ observations and high-resolution models to evaluate climate models and develop improved parametrisations.
- Improved observational resolution of estuaries and coastal regions is needed, to better define their role in basin scale heat and regional freshwater budgets

##### Modelling

A range of model experimentation is needed to inform future monitoring systems. Much of this could potentially be done as part of the emerging TipMIP programme (Tipping Point Model Intercomparison Project, https://tipmip.pik-potsdam.de/), building on recent experiments in the North Atlantic Hosing Model Intercomparison Project NAHosMIP (Jackson et al 2023). Modelling developments include:

- Generating plausible scenarios (storylines) of AMOC tipping, moving from traditional ‘hosing’ experiments to a more direct link with greenhouse gas forcing. This should include scenarios of bifurcation-induced, noise-induced and rate-induced tipping. These scenarios can be used to identify mechanisms of tipping and to develop physically-based early warning indicators.
- Understanding why different climate models have different AMOC tipping behaviour, and identifying circumstances in which the AMOC is likely to tip (either properties of the model climate system, or particular emissions pathways likely to result in tipping). This can inform missions to target key areas of process uncertainty.

##### Data assimilation, reanalysis and prediction

**Fig. 1:**
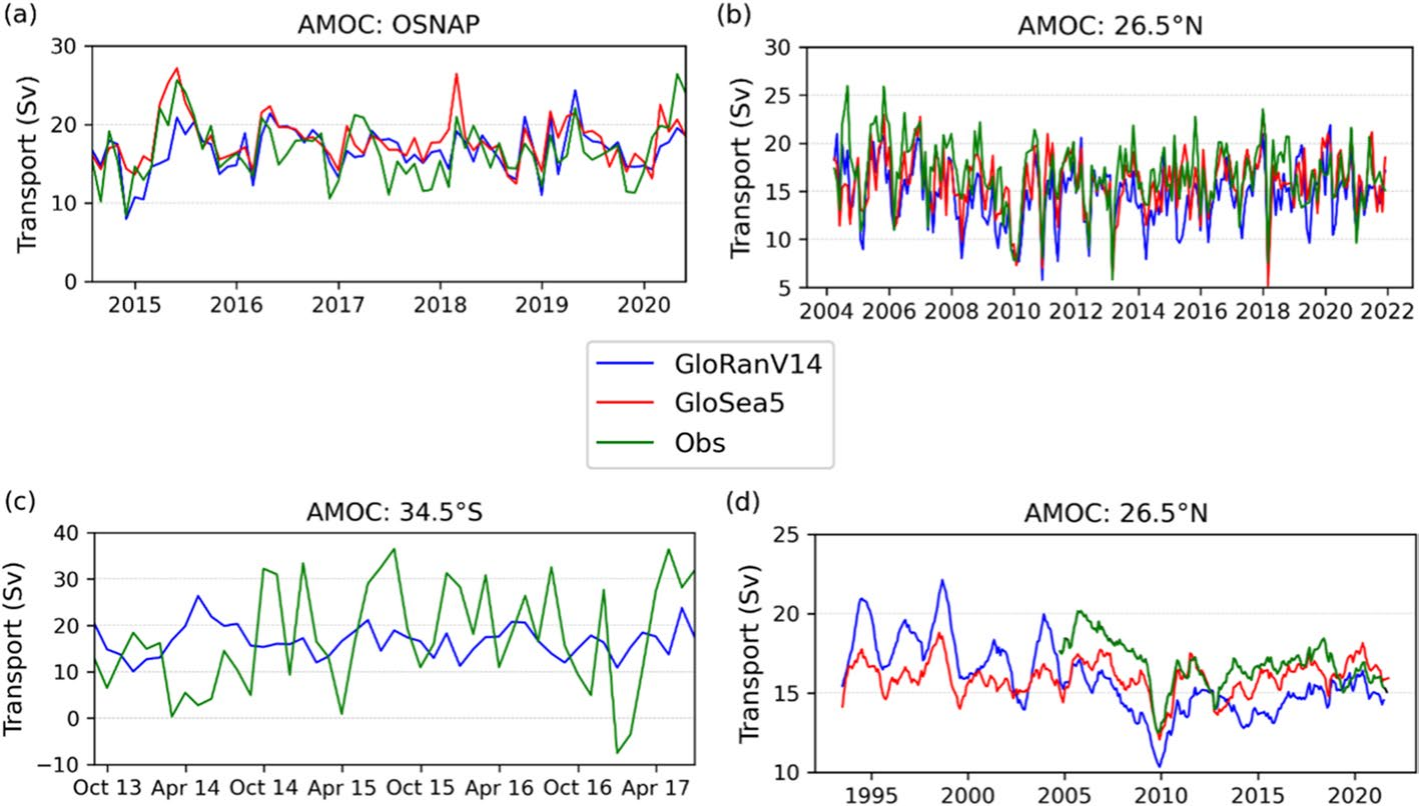
Reconstructions of AMOC from two global ocean reanalysis products: GloSea5 (Jackson et al. 2016, 2019) and GloRanV14 (Baker et al. 2022, 2023), compared with direct estimates on three in-situ sections: **a** OSNAP in the subpolar gyre (Fu et al. 2023a, b), **b** RAPID-MOCHA around 26.5°N (Moat et al. 2023) and **c** SAMBA around 34.5°S (Kersalé et al 2020, 2021). In each case the period shown is that for which direct observations are available. In **c** only the GloRanV14 product is shown, as issues with the altimeter assimilation in the earlier GloSea5 product caused spurious MOC features at this latitude. In **d** is shown the 12-month running mean AMOC at RAPID-MOCHA, back to the start of the reanalysis (1993, coinciding with the start of precision altimetry), illustrating the potential of reanalysis to provide a longer historical perspective on limited in situ timeseries

Assimilation of multiple data types into advanced models (reanalysis) provides a synthesis of observations, which are themselves sparse and incomplete, with physical process understanding. Current reanalysis products capture many aspects of AMOC variability seen at the three trans-basin in situ AMOC sections (Fig. 1), and allow the observations from the sections to be extended back in time, providing a longer historical context (Fig. 1d). However, current reanalyses by no means capture all features seen in the sections. Comparison with the SAMBA (South Atlantic MOC Basin-wide Array) section (Fig. 1c) is quite poor; however, Baker et al. (2023) show that the reanalysis is well correlated with an alternative estimate of monthly-mean AMOC variability on this section, derived from combined altimeter SSH and in-situ hydrographic data (Dong et al. 2021).

Future development may consider the following:

- Assimilation of new data types, e.g. surface salinity measurements provided by SMAP (Soil Moisture Active Passive) and SMOS (Soil Moisture and Ocean Salinity) (Vinogradova et al 2019; Boutin et al. 2023), perhaps also with higher spatial resolution, and improved coverage at high latitudes (Estella-Perez et al. 2020)
- Improved data assimilation methods (e.g. Polkova et al. 2019; Barthélémy et al. 2022), simulators and Observing System Simulation Experiments are needed to make the most of the information in existing EO datasets and guide future missions, e.g. most current data assimilation systems give low or zero weight to altimeter data outside the subtropics, due to the challenge of consistently combining multiple data types, especially in the subpolar region where stratification is weak but dynamically important.
- While AMOC tipping is likely to take longer than decadal timescales, decadal prediction systems, initialised with a wide range of observations, may give early indications of whether observed changes show the beginning of a longer term trend or simply represent random variability.

### Atlantic Subpolar Gyre

#### Nature of Tipping Point

Convection in the Labrador and Irminger seas south of Greenland and the associated Subpolar Gyre (SPG) circulation form a regional ocean circulation element, that is related to the larger scale AMOC, but is not the same thing, as a substantial component of the SPG circulations recirculate regionally without a direct impact on the global circulation. The SPG circulation has been proposed as a climate tipping element with medium confidence by Armstrong McKay et al. (2022). That study was based on analyses of CMIP5 (Sgubin et al. 2017) and CMIP6 (Swingedouw et al. 2021) databases and the IPCC Special Report on Oceans, Cryosphere and Climate Change (SROCCC, Collins et al. 2019a, b). Armstrong McKay et al. (2022) estimated with high confidence that the SPG might have a global warming threshold of ~ 1.8 °C (1.1–3.8 °C) and a tipping timescale of ~ 10 years (5–50 years). The impact of such a collapse of the SPG might be substantial for the climate in the bordering regions (Collins et al. 2019b, a; Oltmanns et al. 2020; Sgubin et al. 2019) but also with wider large-scale teleconnections in the tropical areas, as well as for marine biogeochemistry (e.g. Gary et al. 2020). The Little Ice Age has been linked to weakening of the SPG (Michel et al. 2022) possibly due to large exports of sea ice that followed atmospheric blocking relaxation (Lapointe and Bradley 2021).

Nevertheless, we argue here that there is large uncertainty concerning the risk of this system to tip, given the high dependency of the collapse of the SPG on the model considered. Generally speaking, there are fewer models that do show a rapid collapse of the SPG than ones showing none or very gradual ones. Also, the Arctic and North Atlantic basins exhibit a high degree of variability and complexity, implying interactions between the ocean, the atmosphere and the cryosphere as well as fine-scale processes (Yashayev et al. 2015; Yashayev and Loder 2016; Romanou et al 2023), which even further decrease the confidence we might have in the current generation of climate models. The models showing a tipping are among the best in terms of ocean stratification both in CMIP5 and CMIP6. Stratification is indeed a key variable of ocean dynamics, in particular for what concerns convection, a central element of the SPG. It is at the heart of the positive salt advection feedback arising from this system: if convection ceases in the Labrador and Irminger Seas, the North Atlantic drift is no longer steered into these basins, and the supply of tropical salty waters in these seas is reduced, leading to a strong halocline that further suppresses convection.

This positive feedback can lead to a very stable new steady state with the risk of irreversibility and hysteresis behaviour (Born et al. 2013), which has nonetheless been poorly evaluated in the literature up to now. The threshold of convection is related to surface density, mainly driven by Sea Surface Salinity SSS (Swingedouw et al. 2020; Romanou et al 2023). For a given critical SSS threshold, the temperature cooling in winter is not sufficient to destabilise the water column: the freezing point is reached before convection can occur (as e.g. in the Arctic), so that sea ice is forming in place of convection. As shown in Swingedouw et al. (2020), in CMIP5 models, the threshold might be relatively close to the present-day observed state.

A strong freshwater anomaly is building in the Beaufort gyre from the Arctic, so that there is now considerable risk that this freshwater might flush in the SPG (Zhang et al. 2021, Lin et al. 2023, see Sect. 2.3). Given that the SPG system already recently experienced its largest freshening for the last 120 years in its eastern side (Holliday et al. 2020), this is raising serious concerns.

#### EO Needs and Opportunities

##### Early Warning

The potential proximity to a threshold in SSS in the SPG highlights the need to use models initialised towards observations to include the recent freshening of the North Atlantic—Arctic sector. Such models starting from observed initial conditions constitute the decadal climate prediction systems. More than 10 such systems are developed around the world (WCRP decadal prediction) and provide predictions every year for the next decade (Polkova et al. 2023). Those models are assimilating the most recent EO (typically SST and altimetry) alongside in situ data such as Argo, and are therefore making a good integration of those data into a potentially useful early warning system, but should also integrate new SSS products in their assimilation scheme. The increase in error of SSS products with increasing latitudes is a limitation for data assimilation, which we hope might be improved in the near future. Particular opportunities include:

- Refinement of some components of the radiative transfer modelling used in the salinity retrieval chain, such as the dielectric constant or sea surface roughness modelling (e.g. Boutin et al. 2023)
- Application of new Radio Frequency Interference (RFI) mitigation techniques (e.g. Bonjean et al. 2024), planned to be applied in the future version of CCI v5 SSS.
- Develop corrections for contamination by the proximity of sea ice (Jiménez et al 2021)

In the longer term, the Copernicus Imaging Microwave Radiometer (CIMR), scheduled for launch in 2028, should achieve better radiometric resolution and stability, powerful filtering of RFI-contaminated measurements, and provide multi-frequency measurements that should enable improved corrections of temperature- and wind-related effects.

The use of lower frequencies than L-Band that are more sensitive to SSS in cold waters is another potential way for reducing SSS errors in cold waters (Johnson et al. 2021). The CRYORAD mission idea, recently selected by the European Space Agency ESA, is proposing such a concept. Nevertheless, the decadal prediction systems usually exhibit very large drift when starting from an observed state (Bilbao et al. 2021). This is due to the model biases and the fact that, starting from an observed state, the model usually drifts towards its own mean state. In the SPG, this can be crucial, since CMIP6 climate models usually exhibit a strong cool bias, so that the drift is usually a cooling drift, which might prevent the possibility of predicting a strong cooling that might really occur. The value of bias correcting the forecasts to remove drift, in a case where a tipping point is about to be crossed, is therefore limited. Improvements concerning the initial shock of a decadal prediction system is therefore strongly needed in order to provide skillful prediction of the SPG fate (Polkova et al. 2023). Such improvements might be achieved through reduction of model bias, through assimilation of new data types such as SSS, or through improved methods of assimilating multiple data types into biased models, especially at depth (e.g. Polkova et al. 2019; see also altimetry at high latitudes, see Sect. 2.1).

**Fig. 2:**
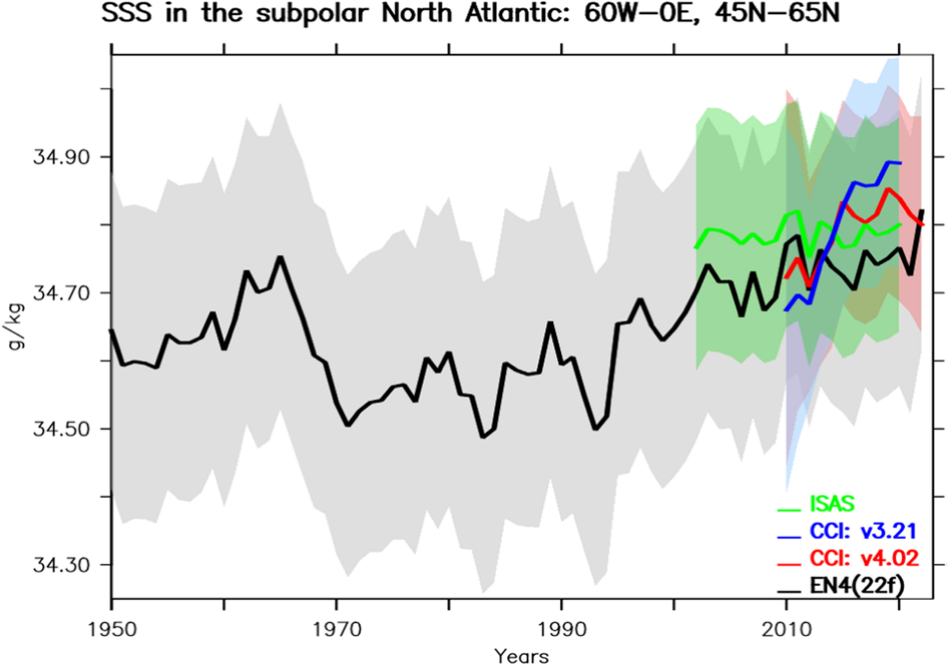
SSS time series since 1950 averaged the subpolar gyre defined as the box 60–0°W, 45–65°N for different monitoring systems. In black is represented the EN4 product and in green the ISAS products, both of which are mainly based on in situ observation records, notably from ARGO from 2004. In blue and red are recent products from CCI in two different versions (Boutin et al.2021a, b). The overlap represents an upper estimate of the error for each product. It is estimated as the annual and spatial mean of the uncertainty provided at the gridpoint level. The mask from CCI v4 products using only gridpoints without sea ice has been applied to all products to allow a correct comparison of the data products

The direct use of EO observations as independent early warning systems might be very relevant for the SPG. In this respect, existing systems that monitor SSS (e.g. SMAP and SMOS, Vinagradova et al. 2019; Boutin et al. 2023) may provide important ground-truth information. The recent SSS data from EO observations (CCI) are shown in Fig. 2 as compared to in situ ones from EN4 and ISAS. The different SSS products are mainly consistent, they do not entirely agree with each other for the recent trends in terms of their best guess. While EN4 and ISAS show a relatively stable SSS evolution in the 2010s, the CCI product exhibits a large increase, mainly due to an upward shift in the years around 2013. This upward shift is reduced with the most recent CCI version which was better filtered for radio frequency interferences even though inconsistencies remain. Understanding and resolving this type of inconsistencies between the observation products might be required to better assess the crossing of a SSS tipping point in the SPG system, which remains also to be better estimated in climate models.

Another avenue of research might be to better analyse models showing abrupt changes in their SPG and the way their patterns in some observable variables evolve just before the collapse. This approach, which can be seen as a space-for-time substitution (Dakos et al. 2010) has been used for the Amazon rainforest (Verbesselt et al. 2016) and might be very promising for the ocean realm. In this respect the SSH, SST and SSS observations might be very relevant and useful, notably given their already relatively high resolution in space (~ 25 km) as compared to CMIP6 models (gridpoints usually larger than 50 km). Observations of the Arctic, in terms of SSS and sea ice cover and transport might be also informative, given the risk of a massive flush of freshwater from the Arctic towards the SPG. In this respect, reduction in the errors of SSS at such very high latitudes (Estella-Perez et al. 2020) should be considered as a key requirement for future EO products.

##### Model Development and Evaluation

Present-day climate models are missing key processes of the SPG dynamics (Le Corre et al. 2020). In particular, CMIP6 models poorly represent the role of eddies, notably for the spread of freshwater anomalies from Greenland Ice Sheet (GIS) melting (Martin and Biastoch 2023, Swingedouw et al. 2021), but more generally concerning water transformation, a key process for stratification properties of the SPG (Desbruyeres et al. 2019; Hirschi et al. 2020). In this respect, next generation models should try to better represent fine scale processes, either through improved parametrization or grid-zooming in some key locations (Hewitt et al. 2020; Zanna and Bolton 2020). The challenge in modelling is compounded by the fact that seasonal sea ice cover in the SPG area plays a crucial role in modifying atmosphere–ocean buoyancy fluxes. Improved resolution of EO products will be decisive in this respect to better assess the dynamics of those processes, and can be used also for spatial early warning, or to evaluate freshening on and off the Greenland coast (e.g. Castelao et al. 2023), relative to EO-based estimates of meltwater from the ice sheet (e.g. Bamber, et al. 2018). Finally, consistent assimilation of multiple EO and in situ data types into models is a major challenge, especially in regions such as the SPG where stratification is weak. Improvements in this area have the potential to extract greater value from the available datasets for modelling and prediction systems.

### Beaufort Gyre Freshwater Accumulation and Release

#### Nature of Tipping Point

The circulation of the upper Arctic Ocean can be schematically characterised by two main features, i.e. the Beaufort Gyre (BG) and the Transpolar Drift (TPD) (Timmermans and Marshall 2020). They represent the sea ice and upper ocean response to the large-scale atmospheric forcing in the Arctic – North Atlantic region dominated by the Beaufort high (BH), accompanied by anticyclonic winds, and the Icelandic low (IL), accompanied by cyclonic winds. The latter represents a large persistent atmospheric low-pressure centre located between Iceland and southern Greenland and it is the main atmospheric driver of the North Atlantic Subpolar Gyre (SPG) discussed in Sect. 2.2. Notably, the North Atlantic storm track originating from the IL has a strong effect on winter weather over Europe and North America (Serreze and Barry 2005) and its northerly shift represents the positive phase of the North Atlantic Oscillation (NAO), which can affect the BG (Moore et al. 2018), hence the BG.

**Fig. 3:**
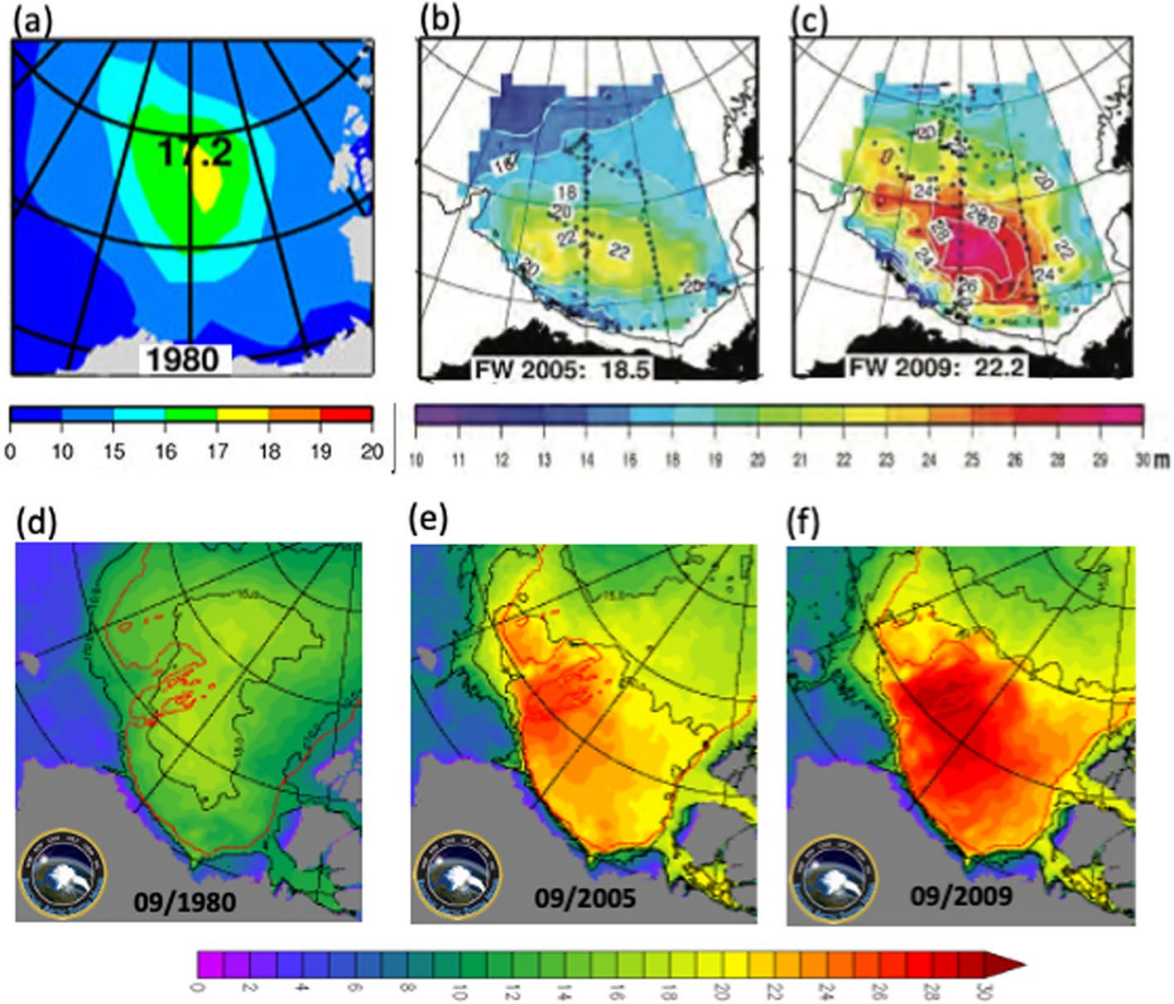
Estimates of the BG freshwater content (in meters) from observations (top; https://www2.whoi.edu/site/beaufortgyre/) and the high-resolution Regional Arctic System Model (RASM; https://nps.edu/web/rasm) (bottom), in 1980 (**a**, **d**), 2005 (**b**, **e**), and 2009 (**c**, **f**)

The BG anticyclonic circulation tends to trap both sea ice and relatively fresh water within, which sets the vertical stratification in the western Arctic (Timmermans and Toole 2023). The sea ice present in the BG for most of the year is not only a source of freshwater in summer but also modulates the wind momentum transfer, which drives the gyre. Its thinning and decline over the past decades (Ding et al. 2017) increase the solar radiation and wind momentum (Rampal et al. 2009) transfer to the upper ocean, hence the intensification of the BG, an increased accumulation of freshwater within (Fig. 3), and a stronger cold halocline (CH, see Sect. 2.4 for more discussion). Estimates of freshwater content in the BG show significant interannual variability superimposed over the overall increasing trend (Proshutinsky et al. 2019). The BG freshwater content over the seasonal cycle varies by approximately 4–5 × 10^3^ km^3^ between maximum in December and minimum in April (Proshutinsky et al. 2009), while it has increased by approximately 6.4 × 10^3^ km^3^ from 2003 to 2018, which represents a 40% increase relative to the climatology of the 1970s (Proshutinsky et al. 2019).

Any significant release of freshwater from this reservoir into the North Atlantic could impact the wintertime deep-water formation and stability of AMOC and SPG, as discussed in Sects. 2.1, 2.2. Export of such “Great Salinity Anomalies” (GSAs) out of the Arctic and its propagation in the subpolar North Atlantic has been observed in the past (Dickson et al. 1988; Belkin 2004), including more recently (Bilo et al. 2022). While the consequent positive buoyancy flux at the surface of the subpolar Atlantic has been shown to freshen subpolar waters produced by deep convection, or to suppress deep convection all together (Dickson et al. 1988; Bilo et al. 2022), its impact on the circulation of the SPG has been less clear. Comparison of the advection rate of the observed GSA events in the 1970s, 1980s, and 1990s, reveals a substantial intensification of the circulation in the SPG (Belkin 2004).

Recent analysis by Lin et al. (2023) of an extensive hydrographic dataset from 2003 to 2019 and satellite-derived dynamic ocean topography data shows that the BG has transitioned to a quasi-stable state over the past decade, as represented by the sea surface height and freshwater content. They also find that the centre of BG has shifted southeastward (also see Fig. 3) due to regional wind forcing, and the CH layer within the BG has thinned significantly. The southeastward movement of the BG moves its freshwater closer to the Arctic gateways to the subpolar Atlantic (Canadian Arctic Archipelago and Fram Strait). If continued, such changes could change the current stable state of the BG and allow a release of freshwater into the Atlantic.

#### EO Needs and Opportunities

The primary hydrographic data on the evolution of the BG and its freshwater content have been provided by *in-situ* measurements as part of the Beaufort Gyre Observing System (BGOS; https://www2.whoi.edu/site/beaufortgyre/). The BGOS, a collaborative project between the Woods Hole Oceanographic Institution (WHOI) in the U.S. and the Institute of Ocean Sciences (IOS) in Canada, was established in 2003 to measure fresh water and heat content / fluxes in the BG using moorings, drifting buoys, and remote sensing, with support from the U.S. National Science Foundation (NSF). In addition to the altimeter and laser measurements of sea surface height, snow, and sea ice thickness discussed in Sect. 2.4 below, measurements of sea ice drift and sea surface salinity derived from scatterometers and radiometers have been helpful to investigate the complex dynamics of the BG. However, their relatively coarse resolution has limited their application to study many important ocean and sea ice processes and interactions occurring at mesoscale (e.g. Manucharyan et al. 2022). Increasingly, km-scale and sub-daily measurements are in demand, in part due to the advancement of regional (Maslowski et al. 2012, 2014; Clement Kinney et al. 2022) and global (Wang et al. 2020; Jeong et al. 2023) high-resolution and process-resolving Earth system models (see Figs. 2, 3, 1) accelerated by the exascale computing capability (https://www.exascaleproject.org/). Such high-resolution observations will allow the complex air-sea-ice interactions in the region to be better quantified, and transports through the narrow gateways between the Arctic and Atlantic basins to be monitored.

### Arctic Halocline Stability

#### Nature of Tipping Point

The vertical stratification in the Arctic Ocean is commonly characterised by a cold halocline (CH) separating a relatively fresh and cold water layer above from the warm and salty Atlantic Water (AW) below (Aagaard et al. 1981). The CH represents a heat sink for the underlaying AW and its strength determined by salinity driven vertical density gradient regulates the rate of upward heat release from AW to the surface mixed layer and sea ice. The stability of CH is critical to the presence of the overlaying sea ice, as it inhibits deep convection and it partly explains why vertical mixing rates in the upper Arctic Ocean away from steep topography are lower than in midlatitudes (Rippeth et al. 2015). The low mixing rates are also attributed to low tidal energy and sea ice limiting the wind momentum transfer from the atmosphere (Dosser and Rainville 2016).

**Fig. 4:**
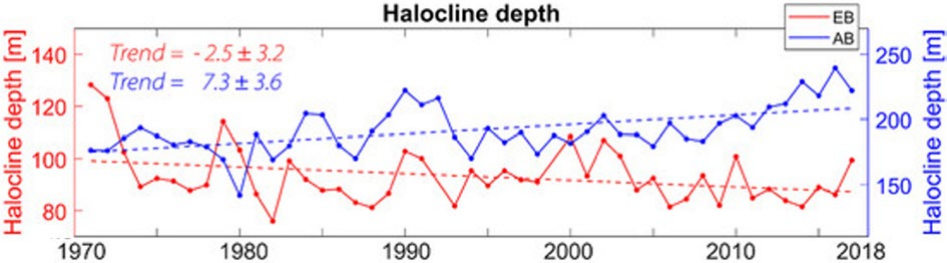
Observed annual mean depth of halocline base in the Eurasian basin (EB; red) and Amerasian basin (AB; blue) regions (adapted from Fig. 1b of Muilwijk et al. 2023)

However, changes in the distribution and strength of the CH layer in the Arctic Ocean have been observed in the past. Comparing the submarine hydrographic data from the Scientific Ice Expeditions (SCICEX) in the 1990s against the long-term climatology, Steele and Boyd (1998) revealed the retreat of CH layer from the Amundsen Basin back into the Makarov Basin. This change was attributed to a weakening of the Beaufort High, which in turn resulted in a cyclonic shift of the sea ice Transpolar Drift and freshwater export from the Siberian shelves into the deep basins of the Arctic Ocean (Maslowski et al. 2001). A more recent analysis of the 2002–2015 mooring data from the eastern Eurasian Basin by Polyakov et al. (2020) has shown weakening and shoaling of CH, which was linked to winter convection due to brine rejection during sea ice formation. The decrease of the CH depth in the Eurasian Basin at the rate of -2.5 m per decade has been recently confirmed by Muilwijk et al. (2023). However, their analysis of observed mean CH depth in the Amerasian basin (or western Arctic) between 1970 and 2018 indicates an opposite, deepening CH depth trend of 7.3 m per decade (Fig. 4). The latter trend could in part be related to the increasing freshwater content in the Beaufort Gyre, as discussed in Sect. 2.3 above, and it emphasises the need to understand critical processes and feedbacks operating in different parts of the Arctic Ocean.

Given the observed summer sea ice retreat over the satellite record (Druckenmiller et al. 2022), a more efficient and direct air–sea coupling is expected to increase vertical mixing, via generation of surface and internal waves (Liu et al. 2016), and the upward heat flux, thus constituting positive wind-ice-ocean feedback (Fine and Cole 2022). Another positive feedback linked to ice-ocean dynamics relates the thinning (Kwok 2018) and reduced strength (Hibler 1979; Rothrock 1975) of the Arctic sea ice to increased ice drift (Gascard et al. 2008; Rampal et al. 2009), ice-ocean stress, and the upper ocean mixing (McPhee 2008), With increased mixing, the heat accumulated in the upper ocean due to summer insolation (Maykut and McPhee 1995) can be entrained into the surface mixed layer (Maslowski et al. 2012) to reduce ice growth in winter and melt more ice in summer. In both cases, the ice-ocean coupling augments the summer-dominated ice-albedo feedback.

Impacts of such dynamically controlled feedbacks have been recently investigated by Beer et al. (2023) using an idealised climate model with varying specified mixing rates for ice-covered and ice-free surface conditions. They find that the wind-ice-ocean feedback can result in a novel bistability in the climate system, which is associated with a hysteresis loop within forced warming and cooling for a limited range of parameters. Furthermore, this study shows that an abrupt tipping point can occur with an irreversible transition to ice-free conditions when crossing the bifurcation point during forced warming. While not conclusive, this study points to the need for improved models of the Arctic and continued and new data to better constrain them and to further investigate potential tipping points associated with the stability of Arctic cold halocline (CH).

#### EO Needs and Opportunities

One example of EO measurements in support of the CH stability is altimeter data from polar orbiting missions, i.e. *ERS-1/2*, *Envisat, and CryoSat-2*, to measure sea surface height, snow, and sea ice freeboard/thickness. Their application to investigation of wave and wind climate in the Arctic Ocean was reported by Liu et al. (2016). However, only CryoSat-2 is still active, while well beyond its design lifetime and likely approaching the end of its mission. Note, that CryoSat-2 measurements of ice freeboard overlapping the NASA IceSat-2 mission, have also allowed improved estimates of snow and sea ice distribution and thickness in the Arctic Ocean (Kacimi and Kwok 2022). A major challenge for EO missions is providing a year-long data on the ocean surface (salinity, temperature, height), sea ice (thickness, deformation, drift) and snow at km and daily-to-weekly timescales. Hence, future EO high spatial and temporal resolution measurements of the Arctic Ocean surface, snow, and sea ice are critically needed.

In parallel, models of the Arctic Ocean and climate need significant improvements to reduce persistent biases, especially in representing the coupling across the air-ice-ocean boundary layer (Maslowski et al. 2012), sea ice thickness distribution (Watts et al. 2021), and the upper ocean vertical stratification (Shu et al. 2023). Some progress is being made in these areas in part due to increased spatio-temporal resolution applied to regional Earth System models (Lee et al. 2023a, 2023b) or global models using unstructured grid with regional refinements (Veneziani et al. 2022; Wang 2024). However, expanded measurements, better understanding, and model representation of critical physical processes, for example involved in shelf-basin exchanges or vertical mixing, are needed and require a combination of in-situ and remote observations.

### The Kuroshio Large Meander—an Anomalous State of a Multi-Stable Western Boundary Current

#### Nature of Tipping Point

Boundary currents are an essential part of the global ocean circulation. Among them, especially western boundary currents like the Gulf Stream in the North Atlantic and the Kuroshio in the North Pacific can exhibit a rich variety of spatio-temporal flow patterns due to their interactions with atmospheric forcing factors in the presence of complex coastlines and bathymetry. At the same time, their pathways impact downstream currents and associated Sea Surface Temperature (SST) patterns (Qiu et al. 2020) with profound impacts on the marine biosphere (Chang et al. 2019; Lizarbe Baretto et al. 2021), ocean–atmosphere heat fluxes and resulting atmospheric processes potentially alternating air temperature, wind and precipitation patterns over large regions.

While for many well-studied parts of such boundary currents (e.g. the Gulf Stream and North Atlantic Current), there seems to exist a continuum of possible pathways, the Kuroshio south of Japan presents a rather unique case with a clear clustering of observed current pathways indicating multistability of the regional ocean circulation system. Specifically, extending previous works already starting in the 1960s, Kawabe (1995) suggested the existence of three distinct major pathways of the Kuroshio termed near-shore and off-shore non-large meander and typical large meander (nNLS, oNLS and tLS, respectively). Especially the large meander state with its recirculation gyre and enclosed cold-surface water anomaly has attracted great interest during the last decades due to its complex dynamics and relevance for the climate and marine biosphere around the Japanese islands. The existence of discrete states opens the possibility of a discontinuous response to external forcing such as climate change; however the research reported here represents attempts to understand these inherently discrete states of the system, which is a pre-requisite for anticipating climate change responses.

Between 1950 and present-day, eight Kuroshio large meander events took place at apparently random occurrence times (Qiu and Chen 2021) and with a broad distribution of lifetimes of the large meander state, the latest being the longest on record and continuing since August 2017. By combining sea-surface height (SSH) observations from satellite altimetry with reanalysis based surface wind stress patterns and comparing the recent patterns with the conditions during the previous (2004–2005) large meander event, Qiu et al. (2023) attributed the unusual stability of the ongoing event to an exceptionally stable dynamic state of the Kuroshio Extension, which minimizes the westward propagation of eddies, potentially destabilising the upstream Kuroshio path while in parallel fostering the maintenance of the recirculation gyre.

##### Impacts

The presence of a Kuroshio large meander has a profound impact on the climate conditions in the Northwest Pacific region around Japan. Sugimoto et al. (2021) reported marked coastal warming during summer off the Kanto-Tokai district of Japan during the present large meander. Using a regional atmospheric model, they could show that a warming of the near-coastal surface waters in the wake of the recirculation gyre leads to an increase of water vapour in the low-level atmosphere due to enhanced evaporation near the coast. Along with altered near-surface winds this results in a net increase of downward longwave radiation over the Kanto region and, hence, increasingly hot and humid summers. By contrast, for winter conditions during the large meander event of 2004–05, Xu et al. (2010) already reported reduced winds and precipitation resulting from the cool water pool between the Kuroshio and the Japanese coast. In general, the cool water pool along with coastal warming off the Tokai province of Japan appears as a common fingerprint of large meander events (Sugimoto et al. 2019). Other related effects of the wintertime presence of a Kuroshio large meander include a southward shift of primary extratropical cyclone tracks along with reduced latent heat fluxes south of Japan (Nakamura et al. 2012; Hayasaki et al. 2013). Based on regional climate modelling, Murazaki et al. (2015) showed that the cold SST anomaly of the large meander consistently leads to reduced upward surface turbulent heat flux, lower precipitation frequency, and less frequent steep horizontal gradients in equivalent potential temperature over the ocean during both summer and winter.

In addition to its pivotal effects on regional atmospheric processes, the presence of a Kuroshio large meander also exerts direct influence on ocean dynamics, resulting in distinct patterns of eddy kinetic energy, potential vorticity, relative vorticity, and eddy-mean flow interaction south of Japan (Ma 2019). Based on a new multi-scale statistical analysis tool, Yang and Liang (2019) identified a spatially coherent inverse cascade of kinetic energy from synoptic eddies to the slowly varying mean flow over the whole large meander region, along with a particular relevance of an initial influx of mesoscale eddy energy from upstream regions responsible for the emergence of the inverse cascade. In another study, Qiu and Chen (2021) identified intense anticyclonic eddies emanating from the Subtropical Counter-Current interacting with Kuroshio path perturbations southeast of Kyushu, generating positive relative vorticity along the coast finally leading to the formation of the large meander. Not surprisingly, the reported effects on ocean circulation at various spatial and temporal scales can also have a profound impact on biological and biogeochemical processes in the Northwest Pacific (Hayashida et al. 2023).

#### EO Needs and Opportunities

From the aforementioned results, it may be noted that especially the turbulent flow patterns in the adjacent Pacific Ocean south of Japan are crucial for understanding the processes leading to the formation and maintenance of the Kuroshio large meander. While atmospheric impacts can be well traced with state of the art in situ and remote sensing based products, the importance of upscale kinetic energy transfer from synoptic scales (Yang and Liang 2019) points to a particular area high-frequency EO data (SST, sea surface height SSH, and potentially SSS, on timescales shorter than 15 days, and especially in near-coastal regions) is important to fully quantify the dynamics preceding the emergence of a large meander. Based on such improved real-time observational datasets along with better process understanding (generated by exploiting physics-based models and/or modern artificial intelligence methods), forecasts of future state transitions of the Kuroshio path and its regional climatic impacts could potentially be developed at societally relevant lead times.

### Ocean Deoxygenation

#### Nature of Tipping Point

With progressing global warming there is an increased risk that climate-relevant biogeochemical ocean elements might cross critical thresholds and tip into novel states, with severe consequences for the climate system, marine ecosystems and human societies (Heinze et al. 2021; Rockström et al. 2009; Lenton et al. 2008). Among the biogeochemical elements that may undergo large and possibly irreversible (on human time scales) changes in response to the ongoing anthropogenic perturbations is the ocean oxygen (O_2_) content, with potentially large effects on marine biogeochemistry and ecosystems (Watson 2016; Rockström et al. 2009; Lenton et al. 2008; Heinze et al. 2021). The oceanic O_2_ content has undergone large variations throughout the earth’s history, driving the evolution of novel metabolic pathways that shaped ocean biogeochemistry as we see it today (Canfield et al., 2010). Dissolved O_2_ is necessary for sustaining aerobic life in the ocean and regulates redox-sensitive biogeochemical processes. The cycling of O_2_ is strongly linked with the nitrogen (N), phosphorus (P), iron (Fe) and the carbon (C) cycles. Changes in oxygen will impact the cycling of any of the other elements and can potentially lead to an amplification/reduction of the initial perturbation, with stabilising or destabilising feedbacks to the climate system.

Currently, ocean warming is affecting the oceanic dissolved oxygen content. Oxygen concentrations have decreased by about 2% over the last 50 years (Ito, et al. 2017; Schmidtko et al. 2017), a process termed ocean deoxygenation. Tropical Oxygen Deficient Zones (ODZs), where oxygen concentrations fall below values critical for the onset of the anaerobic microbial processes and/or have sublethal effects (hypoxia) on animals, are expanding (Stramma et al., 2008). These oxygen changes are due to a combination of chemical, physical and biological drivers (Bopp et al. 2017; Oschlies et al. 2018) and show a significant regional and depth variability (Ito et al. 2017). The reduction in temperature-dependent O_2_ solubility is well quantified and estimated to account only for about 15% of the observed oceanic global O_2_ loss (Helm et al. 2011). The contribution from changes in circulation and associated reduced ventilation is less certain, potentially explaining the majority of the deep ocean O_2_ changes (Breitburg et al. 2018). O_2_ consumption due to biological respiration is reduced in the low-latitude nutrient-limited regions that experience reduced productivity, but enhanced in high-latitude regions where biological production has intensified under global warming (Laufkötter et al., 2015, 2016; Steinacher et al. 2010).

Next to uncertainty of driving processes, detectability of climate-driven O_2_ trends is challenged by natural short-term variability of oxygen (Frölicher et al. 2009; Long et al. 2016; Bopp et al. 2017) and poor spatiotemporal coverage of O_2_ observations (Grégoire et al. 2021). The time required for anthropogenically forced signals to emerge above background noise (time of emergence) is found to be regionally variable. Longer timescales are required in regions where processes cause compensating effects (compensation of the thermal and non-thermal (biological productivity and ventilation) changes), with a resulting net weak trend (Bopp et al. 2017) such as in the tropical regions (Cabre et al. 2015). Model projections suggest that while stopping CO_2_ emissions will stop ocean surface warming and near surface ocean deoxygenation, the deep ocean layers are expected to continue losing oxygen for centuries (Oschlies et al. 2021; Frölicher et al. 2020). This committed deoxygenation results from the reduced solubility of warm deep waters that have been last in contact with the atmosphere during the high CO_2_ emission era, and the longer ventilation ages in the ocean interior due to the weakened overturning circulation inertia (Oschlies et al. 2021). However, high uncertainties exist if current steady ocean deoxygenation can evolve into abrupt widespread O_2_ loss over human timescales scales (centennial).

Paleoclimate records indicate that widespread ocean deoxygenation events, known as Oceanic anoxic events (OAEs), associated with profound geochemical changes, occurred multiple times during the Earth’s history (Jenkyns 2010). The occurrence of these events has generally been associated with an abrupt temperature rise induced by rapid atmospheric CO_2_ increase (e.g. by volcanic activity) and associated reduced O_2_ solubility, ocean circulation changes and, on time scales longer than 100 kyears, enhanced continental nutrient weathering (Kiehl and Shields, 2005). It is hypothesised that amplification by positive biogeochemical feedbacks such as phosphorus (Jenkyns 2010; Monterio et al., 2012; Watson et al. 2017) or iron (Fe) sediment regeneration (Wallmann et al. 2019), from low-oxygen sediments, is required to further boost the biological demand of oxygen, and meet the conditions for wide-spread OAEs. Characteristics of localised ODZs (nowadays in the tropical low latitude ocean basins) have changed over time. ODZs expanded and contracted not only over millennial but also on shorter, centennial and decadal timescales in response to ocean ventilation changes, due to climate fluctuations (Jaccard and Galbraith 2012; Galbraith et al., 2013) and natural variability (Stramma et al. 2020; Segschneider et al. 2018), and ecological shifts that control the depth of organic particle remineralization (Lu et al. 2018, Hülse et al. 2021; Crichton et al. 2023). Continental configuration is also thought to influence the sensitivity of the ocean system to biogeochemical feedbacks (Monteiro et al. 2012, Meyer et al. 2008).

Currently the drivers, the sign of change and time scale of O_2_ tipping are not well known (Armstrong McKay et al. 2022). The projected O_2_ decline under global warming scenarios is found to rebound within several thousand years in multi-millennial model studies due to circulation reinvigoration (Yamamoto et al., 2015; Frölicher et al. 2020) or to lagged responses in biogeochemical feedbacks (Oschlies et al. 2019; Kvale et al., 2019). O_2_ tipping behaviour has emerged in modelling studies associated with the weakening of the overturning circulation, under past atmospheric and/or orbital forcings over millennial timescales (Pohl et al. 2021; Segschneider et al. 2018; Brown and Galbraith 2016; Schmittner et al. 2007), changes to continental configuration (Pohl et al. 2022), and from complex biogeochemical feedbacks, both in box-models (Watson et al. 2017, Wallmann et al., 2003, 2019, 2022; Handoh and Lenton 2003) and in intermediate-complexity Earth system model (ESM) experiments (Kemena et al. 2019; Niemeyer et al. 2017, Lu et al. 2018, Meyer et al. 2008, Hülse et al. 2021, Crichton et al. 2021, Frölicher et al. 2020.). However, a comprehensive assessment of the potential physical and biogeochemical drivers of tipping behaviour in ocean oxygen is lacking. This is mostly due to the limited knowledge of key biochemical complexity and elevated computational costs of long-times scale (centennial to multi-millennial) modelling experiments. As such, our understanding and ability to identify the potentially tipping behaviour of O_2_ is currently limited.

##### Drivers and Timescales

**Fig. 5:**
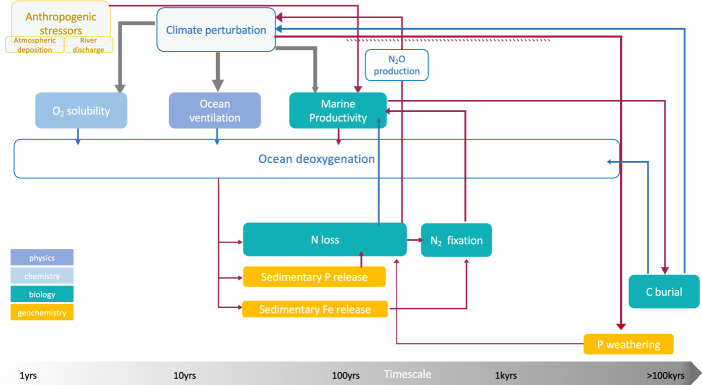
Schematics of the physical, chemical and biogeochemical processes that interact and impact ocean oxygen content over multiple timescales. Red arrows represent positive feedbacks, blue arrows represent negative feedbacks. The anthropogenic climate perturbation affects O_2_ solubility, ventilation and biological O_2_ consumption (grey arrows) potentially with positive or negative feedbacks. With warming O_2_ solubility is reduced and, via changes in stratification and mixing, ocean ventilation declines. The decline in surface nutrient supply due to enhanced stratification affects marine productivity generally with a decline in the tropics and increase in the high latitudes. The net effect is uncertain and may lead to expansion of oxygen deficient zones on decadal to centennial timescales, where anoxic microbial processes, denitrification and anammox, can lead to N loss and the production of the greenhouse gas N_2_O. A negative feedback can occur if N loss is not compensated for by N_2_ fixation, with the potential collapse of marine productivity O_2_ can accumulate as organic matter respiration is reduced. However,  if N_2_ fixation is promoted by sedimentary release of P and Fe and/or P weathering, a self-sustained positive feedback can occur, potentially leading to N runaway and eventually the establishment of euxinia

More than 99% of all of the molecular oxygen on the planet is in the atmosphere with less than 1% being dissolved in the ocean (Oschlies 2019). The oceanic O_2_ content is supplied by uptake from the atmosphere at the surface ocean, mostly driven by O_2_ temperature-dependent solubility and ocean circulation that transports cold surface O_2_-rich waters into the ocean interior during deep water formation at high latitudes. Dissolved O_2_ is lost in the ocean by biological consumption via biological respiration. Factors that affect O_2_ solubility, ocean circulation and biological oxygen consumption can drive ocean deoxygenation (Fig. 5) and trigger O_2_ tipping behaviour.

##### Physical–Chemical Drivers

In model simulations a general pattern emerges on millennial timescales that is consistent with paleoproxy data. Under cold climates, such as the Last Glacial Maximum, the reduction in deep water formation leads to an increasingly separated well-oxygenated upper ocean and a poorly ventilated oxygen-depleted deep ocean (Jaccard and Galbraith 2012; Somes et al. 2017; Pohl et al. 2021). Under warming climates, ocean surface warming leads to a reduction of O_2_ solubility and to the intensification of stratification and slow-down of circulation and vertical mixing, resulting in subsurface ocean deoxygenation and widespread oxygen depletion (Tsandev and Slomp 2009; Ruvalcaba Baroni et al. 2014).

##### Biogeochemical Feedbacks

It is still a matter of debate whether the climate forcing exerted by volcanic CO_2_ emissions, was sufficient to explain the aerial extent of ocean anoxia during past OAE or if additional feedbacks were required to amplify the deoxygenation trend induced by global warming (Jenkyns 2010; Wallmann et al. 2019). Phosphorus is a key nutrient for supporting biological production. This nutrient is generally provided to the ocean by continental weathering and is removed from the ocean by being incorporated in marine sediments. However, if bottom water O_2_ concentrations fall below a certain threshold level this element can be released from sediments back into the water (Van Cappellen and Ingall 1994), where it can fertilise organic matter production and remineralization (Beil et al. 2020; Jenkyns et al. 2010; Watson et al. 2017). Despite large uncertainties regarding the weathering and sedimentary P fluxes model parameterizations, recent modelling studies have shown that enhanced ocean P fluxes from weathering and/or from anoxic sediments, under present, future (Niemeyer et al. 2017; Kemena, et al. 2019) and past climate scenarios (Wallmann, 2005; Monteiro et al., 2012), can promote additional ocean productivity. This in turn, exerts a positive feedback on deoxygenation, leading to a large increase in the volume of ODZ on millennial timescales. The large expansion of suboxia then causes a net loss of bioavailable N that is consumed during organic matter remineralization in ODZ in the process of denitrification. Eventually, in a negative feedback, a considerable amount of P is left unused in the ocean as N becomes the primary nutrient limiting biological productivity (and is not compensated for by the Fe-limited N_2_ fixers) thus ultimately constraining the expansion of ODZ (Kemena et al. 2019). Hence, although P addition to the ocean drives additional deoxygenation, the positive redox-related feedback on benthic P release is eventually limited by the availability of N and the apparent inability of N_2_ fixers to respond to the larger P inventory. Further, the inability of N_2_ fixers to respond to denitrification allows, in current models that tend to suppress N_2_ fixation in low temperature regions, for a net ocean O_2_ accumulation in a multi-millennial Earth System Model (ESM) simulation (Oschlies et al. 2019). Models without the full representation of the N-cycle feedbacks, miss this important negative feedback limiting global deoxygenation (Wallmann 2003).

On the other hand, how a full dynamic iron cycle in an Earth system model will affect the utilisation of the added P by N_2_ fixers remains to be tested. In such a model setting, primary production is not limited by N-loss and widespread deoxygenation can be self-sustained (Landolfi et al. 2013). Positive feedbacks between sedimentary P and Fe release and ODZ intensification could potentially drive a widespread and lasting expansion of oxygen-depleted ocean regions on centennial timescales, eventually leading to euxinic events (Canfield 2006). This is shown in a recent regional box-model study of the Eastern Tropical Pacific. Under current warming conditions if the Fe-demand of N_2_ fixing organisms can be met by O_2_-dependent sedimentary Fe release, a positive feedback loop develops on decadal timescales and amplifies O_2_ decline (Wallmann et al. 2022). As N losses are not compensated for by N_2_ fixation due to stoichiometric constraints (Landolfi et al. 2013), NO_3_ and NO_2_ can become depleted and sulfidic conditions may eventually occur (Wallmann et al. 2019, 2022). The production of hydrogen sulphide (a gas toxic to animals) may be limited by the consumption of sulphide-oxidising photoautotrophs (Meyer et al. 2008) and by the scavenging of it by organic particles, which can help to reoxygenate the ocean after an OAE and reduce atmospheric CO_2_ concentrations by efficiently sequestering biological carbon and providing an alkalinity source (Hülse et al. 2019). The release of H_2_S in euxinic conditions is supported by paleorecords and remains a suspected mass extinction mechanism (Meyer et al. 2008). The potential for negative H_2_S feedbacks to prevent widespread deoxygenation of the water column is not well constrained.

It is unclear whether the burning of fossil fuels combined with human activities delivering additional nutrients, from atmospheric deposition and/or river-discharge and land-use changes, are sufficient to trigger such positive biogeochemical feedbacks (e.g. from increased stratification and associated reduced interior ocean ventilation, in interplay with increased phosphorus input from fertilisers-use, accelerated weathering, land-use changes, and/or recycling from sediments under anoxic conditions), if they could lead to a widespread deoxygenation and over which timescales. Given the large uncertainties over drivers, mechanisms, time-scale and critical thresholds for triggering a widespread oxygen loss, ocean deoxygenation is currently regarded as an uncertain potential tipping element and has not been included within the core tipping elements by the recent study of Armstrong McKay et al. (2022).

What is more, ESM models predict only about half of the ocean deoxygenation that is currently observed (Oschlies et al. 2018), suggesting that current models may lack key physical and biogeochemical feedback mechanisms that amplify the respiratory oxygen demand in the ocean interior (Oschlies et al. 2018). Models that include positive feedbacks between anoxia, phosphorus and/or Fe recycling from sediments and marine productivity have been shown to exhibit a non-linear behaviour, tipping into widespread deoxygenation on centennial to millennial timescales (Kemena et al. 2019; Walmann et al. 2019; Somes et al. 2017). These studies emphasise the critical role of biogeochemical feedbacks in affecting the ocean O_2_ content that need to be accounted for in models for past and future projections.

##### Biological-Ecosystem Impacts of Tipping

Ocean deoxygenation poses a serious direct threat to life in the ocean. Under low oxygen conditions (< 62 mmol m^−3^, hypoxia) metabolic processes such as respiration, reproduction and movement may be impaired (Rabalais et al. 2010). At ecosystem level this will lead to changes in community structure towards hypoxia-tolerant species of fish and invertebrates, habitat compression, migration and mass mortality (Breitburg et al. 2018). Species extirpation (local species loss) can occur when ecophysiological tolerances of temperature and O_2_ threshold are crossed. These can be quantified from the local O_2_-supply to demand ratio (Penn et al. 2019). On a global scale, species extinction can occur when habitat loss exceeds a critical ocean fraction. In a model study extirpation risk has been found to be higher in the high latitude and the tropics under a high emission scenario. Extirpation, an ecological tipping point, is likely to culminate in a mass extinction similar to those in Earth’s past by the end of the twenty-first century under continued global warming (Penn and Deutsch, 2022).

##### Biogeochemical-Climate Impacts of Tipping

The expansion of ODZ (< 5 mmol m^−3^, suboxia) will restrict aerobic remineralization and trigger anaerobic microbial processes, which affect the cycling of elements nitrogen, phosphorus, iron, sulphur and various trace metals, and the production of greenhouse gases such as nitrous oxide (N_2_O) and methane (CH_4_). Under low O_2_ conditions, the anaerobic microbially-driven remineralization of organic matter will proceed consuming nitrate, manganese and iron oxides and sulphate sequentially. The consumption of NO_3_ via the microbial processes of denitrification and anammox can increase. These microbial processes result in the loss of fixed N, that if compensated regionally for by N_2_ fixation may lead to positive feedbacks and self-sustained ODZ expansion (Landolfi et al. 2013) and euxinia may develop (Canfield 2006). During denitrification and nitrification under low O_2_ conditions, the production of N_2_O occurs (Ward and Jansen, 2014). This potent greenhouse gas, if released to the atmosphere, has the potential to amplify global warming and deoxygenation, constituting a positive feedback. Ocean deoxygenation also leads to major changes in the redox-sensitive cycling of essential elements and metals such as P and Fe. Sediment release of these elements can occur under low bottom water oxygen concentrations (Van Cappellen and Ingall 1994; Dale et al., 2015), potentially stimulating organic matter production and remineralization, thereby also exerting positive feedback on deoxygenation (Walmann et al. 2019).

#### EO Needs and Opportunities

##### Monitoring

Since ocean deoxygenation can cause severe ecosystem disruptions and feedbacks to other elements and climate, the availability of early warning systems, in open and coastal ocean is highly desirable. The identification of changes and possible TPs in ocean O_2_ requires adequately long observational records with adequate spatial coverage. Although novel technologies have largely expanded the O_2_ observing system (Grégoire et al. 2021), with O_2_ sensors routinely deployed on a range of in situ platforms (such as ARGO, AUVs and gliders, moorings) that allow the assessment of intraseasonal, seasonal and interannual oxygen variability, challenges remain for identifying long-term trends. The use of EO observations such as temperature (SST), ocean productivity (Ocean Color), combined through artificial intelligence to reconstruct oxygen changes, can provide novel avenues towards monitoring and early warning systems over the satellite era. Of elevated interest for this purpose is the remotely sensed data of chlorophyll a (Chla) a phytoplankton biomass proxy, particulate back scattering (bbp) a proxy for non-algal particulate material, a the derived quantity particulate organic carbon (POC) and their 3D reconstruction, and more widespread availability of HYPER-spectral remote sensing reflectances (Rrs), complemented with field measurements and new in situ observing systems (like BGC-Argo) of key variables such as O_2_ and nutrients. Of crucial importance are also observational constraints on ocean circulation and its changes over time. Abiotic transient tracers can be particularly valuable to provide information about changes in ventilation and residence times over which respiratory signals can accumulate in the ocean interior. Detection of fingerprints of ocean deoxygenation such as N_2_O and H_2_S fluxes at the sea surface via remote sensing (Infrared Atmospheric Sounding Interferometer, IASI) also represent potential avenues that require further development (Garcia et al. 2018).

##### Paleoproxies

The reconstruction of regional O_2_ levels of the past ocean relies on the indirect evidence of O_2_ dynamics from local sedimentary proxy data such as benthic foraminifera, redox-sensitive elements, and isotopic fingerprints. Further efforts towards expanding sparse paleoproxy datasets, improving the resolution of paleo data, providing uncertainty estimates in the paleo-reconstructions are required to advance our quantitative understanding of OAE and attribution of their drivers and enhance our predictive capabilities.

##### Modelling

ESMs are powerful tools to explore the possibility of ocean O_2_ tipping over a range of timescales and forcings. The drivers, location and likelihood of tipping are sensitive to emerging physical and biogeochemical feedbacks (Fig. 5). However, biogeochemical processes are poorly constrained and are not fully represented in current state-of-the-art models. A multimodel intercomparison experiment which included key biogeochemical processes (Fig. 5) would be desirable to test mechanisms of oxygen tipping in models of different complexities. This effort could potentially build from the TipMIP programme (https://tipmip.pik-potsdam.de/), and specifically designed to tackle the following questions:

- What are the drivers, key physical processes and biogeochemical feedbacks, associated with oxygen tipping behaviour?
- What are the characteristic spatial and temporal scales of positive and negative feedbacks and what is their combined effect on ocean oxygen?
- What are the drivers’ tipping points and thresholds and what are their corresponding uncertainties?
- What is the risk of crossing individual TPs, at different levels of ongoing climate and land-use change, which might trigger oxygen tipping behaviour?
- Are the respective O_2_ changes abrupt and on which timescales are they reversible?
- How is the overall stability of the Earth system affected by marine deoxygenation?

Remotely sensed data can further help to constrain biogeochemical processes in climate models, thereby reducing uncertainties in the thresholds of drivers and timescales of climate tipping elements.

Overall, our current scientific knowledge on the tipping behaviour of ocean oxygen is limited, a systematic analysis, drawing from expanded current and paleoproxy observations and multimodel ensemble studies, is desirable. Further development may consider: (i) Expanding spatial and temporal coverage of multiplatform observations of O_2_ and associated variables, particularly those informing about ocean ventilation and changes in ventilation (e.g. abiotic transient tracers); (ii) Testing the use of artificial intelligence (AI) tools to exploit ever growing EO-observations for O_2_ studies (iii) Expanding the sedimentary record and using multivariate approaches for attribution studies (iv) Testing mechanisms of oxygen tipping in models of different complexities.

In addressing these questions, critical knowledge gaps in the oceanic O_2_ controls will be reduced and understanding of Earth system homeostasis will be improved.

## Tipping Points in Pelagic Marine Ecosystems

In the dynamic expanse of the ocean, pelagic organisms, inhabitants of the water column, exhibit continual movement—both vertically and horizontally—engaging in drifting, swimming, and migrating activities. Consequently, conventional time series measurement systems conducted over fixed grids, such as repeated transects, only offer an approximate representation of the temporal dynamics of observed species. The inherent limitation lies in our fixed spatial sampling scale, which fails to align with the fluid variability of the samples. Organisms may be missed during sampling, yet reside slightly deeper or a few metres or kilometres away in any direction, and their presence can be overestimated because of their typically patchy distribution. Moreover, the uncertainty extends to the entities being sampled—be it individuals, populations, or species—making it challenging to ascertain whether the population measured at time 't' is the same as that observed at time 't + n'.

Consequently, there exists a significant contrast between time series studies of pelagic organisms freely moving in three dimensions in the water column, and benthic organisms inhabiting the seafloor and moving in two dimensions with reduced mobility. The former incorporate spatial and temporal uncertainties that are challenging to measure accurately, while the latter provides relatively precise spatial and temporal measurements, particularly for sessile species, offering a degree of population certainty.

Despite numerous studies addressing sampling design and uncertainty in each compartment (Pepin and Helbig 2012; Lin et al. 2010; van Hoey et al. 2019), to our knowledge the inherent difference between these compartments has not been thoroughly assessed.

There has been extensive research on marine benthos tipping points and phase shifts (this is the term commonly used in benthos research, while regime shifts or abrupt shift are the terms commonly used in water column research). Thus, the challenges faced by coral reefs, a global concern among ecologists, are well-documented (Hughes 1994; Matz et al. 2018). Numerous studies explore phase shift and tipping point dynamics leading to the transition of coral reefs to alternative algal states, and several provide future projections, and reef-danger warnings based on earth observations (Nim et al. 2010; Claar et al. 2020; Hobday et al. 2023; Wernberg et al. 2023; Melet et al. 2020; Heron et al. 2016). Due to their role as biodiversity hotspots, nursery grounds, nutrient cycling hubs, and CO_2_ sequestering agents, coral reefs are considered a 'tipping element' in various works (Lenton et al. 2008; Armstrong McKay et al. 2022; Wang et al. 2023).

In contrast, the pelagic ocean organisms, invisible and un-gridded, moving and mixing in three dimensions, have not yet been recognised as a tipping element. The 3D moving matrix complicates the identification of pelagic ecological shifts (often referred to as abrupt shifts), which are not marked by clear irreversibility, a crucial concept in defining tipping points. Nevertheless, shifts in these interconnected and interchanging systems can have potential global-scale consequences, and research has indicated that pelagic shifts can synchronously occur in distant oceanic basins and that the frequency and magnitude of the shifts may increase as the earth system continues to warm (Beaugrand et al 2015, 2019).

This section, therefore, concentrates on abrupt shifts and tipping points in the pelagic components of the ocean, emphasising the potential of EO to monitor their temporal and spatial dynamics and provide early warnings. All pelagic components described in this section exhibit threshold behaviour and possess the potential to impact biological elements (such as the food web) and even contribute feedback to the climate (e.g. phytoplankton), making them 'invisible' tipping elements.

The pelagic component, particularly the plankton community, plays a vital role in sustaining life on Earth. Phytoplankton, forming the foundation of marine food webs, contribute to approximately half of the global primary production. Additionally, through various processes and interactions (e.g. carbon sequestration, biological pump, aerosol/DMS production, ocean albedo, oceanic uptake of heat), they influence temperature, ocean acidity, carbon cycling, and the overall climate system (Miloslavich et al. 2018). Marine zooplankton, sustained by phytoplankton, encompass a diverse range of species, including copepods, krill, and jellyfish, along with the larval stages of most marine animals. Each plays a specific role in nutrient cycling and ecosystem stability, holding immense ecological and economic importance in marine ecosystems. Their impact extends to human societies, significantly contributing to multiple economic sectors. Fisheries and farmed seafood, sustained by plankton, provide a significant source of animal protein to more than 3 billion people globally, with the total first sale value of global production estimated at USD 406 billion in 2020 (of which USD 141 billion for capture fisheries and USD 265 billion for aquaculture, FAO 2022).

Presently, human activities exert a profound impact on marine life at scales ranging from global to local, including global warming, acidification, deoxygenation, overfishing, pollution, and notably, the burgeoning issue of plastics. If current trends persist, the weight of plastics in the world's oceans is projected to surpass that of fish by 2050 (Industry Agenda 2016). All these factors, individually or in combination, can propel marine communities towards ecological shifts.

The *pelagic tipping point dilemma* thus revolves around the vital task of identifying tipping points and ecological shifts in a vast, dynamic ecosystem. The inherent challenges stem from its vastness, spanning regional to semi-global scales, its dynamic 3D nature, and its interconnectedness with other biological and physical components of the Earth system. Thus, the potential consequences of tipping points in the pelagic ecosystem are far-reaching, surpassing the confines of specific habitats or areas. Early warnings of hard-to-identify critical changes (including dimension, intensity and pace of the changes) are consequently *crucial* and *complex* because of the inherent mismatch between sampling design and sampled population.

Satellite observations offer a promising avenue in addressing this dilemma. Their extensive scope and comprehensive coverage make a substantial contribution to this quest. These observations provide consistent, and in some cases decade-long, monitoring on a global scale. While they may be limited in the depth dimension, satellite observations excel in providing excellent horizontal coverage. This capability accommodates regions with diverse requirements, resources, and monitoring capabilities, offering valuable insights into the elusive open ocean.

### Phytoplankton Productivity and DMS-Phytoplankton-Cloud Interactions

#### Nature of Tipping Point

##### Unpredictable Abrupt Shifts During the 21st Century

Marine phytoplankton, constituting half of the global net primary production, play a pivotal role in sustaining the Earth's Biosphere (Field et al. 1998). Their productivity and diversity in the ocean control the bottom-up dynamics of crucial commercial seafood products, influencing the magnitude and pathways of energy transfer within the marine food web. In a prior review on tipping elements in marine ecosystems (Sect. 2.2 of Swingedouw et al. 2020), the remote sensing of chlorophyll-a for monitoring phytoplankton was recognised as an advanced and accessible Earth Observation tool for marine ecosystems.

Satellite ocean colour sensors have continuously provided information on the global distribution of surface phytoplankton biomass and community composition since the late 1990s (Groom et al. 2019). This information has unveiled global-scale changes, including the expansion of the oligotrophic ocean (ocean desertification) and phenological shifts in seasonal blooms which can have substantial consequences for higher trophic levels (Sect. 2.2.4 of Swingedouw et al. 2020).

A notable recent application of remote sensing to phytoplankton involves the development and use of an advanced lower trophic level marine ecosystem model. This model incorporates 35 phytoplankton groups based on various functional types and size classes (Dutkiewicz et al. 2021), offering a more comprehensive representation of phytoplankton diversity compared to current-generation Earth System models, which typically include only 1–3 phytoplankton groups (Kearney et al. 2021). Advances in ocean colour sensors and algorithms have allowed the estimation of sea surface chlorophyll-a concentration for different size classes (e.g. Ward 2015), contributing to a better understanding of the global distribution of the simulated phytoplankton community structure in recent decades (Dutkiewicz et al. 2021).

A global climate projection utilising the 35-compartment phytoplankton model under a high greenhouse gas emission scenario illustrates potential abrupt shifts in phytoplankton biomass, productivity, and community structure across various oceanic regions during the latter half of the twenty-first century (Cael et al. 2021). These shifts may exceed 50% relative to historical baselines, covering up to 25% of the tropics and subtropical gyres (Cael et al. 2021). Notably, these abrupt shifts are anticipated on species requiring specific resources like diatoms, dinoflagellates, and diazotrophs (Dutkiewicz et al. 2020), particularly in subtropical regions (Cael et al. 2021). Importantly, these shifts occur independently of gradual changes in environmental variables, such as temperature and nutrients, making them unpredictable using standard early warning signals (Cael et al. 2021).

The causes of these abrupt shifts are unclear, but their occurrence at the edges of subtropical gyres suggests a potential link to the expansion of ocean deserts (Cael et al. 2021; Karl et al. 1995). Whether these shifts are reversible or not remains an open question that requires projections beyond the twenty-first century. Nevertheless, such abrupt shifts could trigger cascading effects in higher trophic levels and biogeochemical cycles due to the strong coupling of these systems. Investigating these consequences can be achieved by incorporating outputs from phytoplankton diversity models (e.g. Cael et al. 2021) into higher trophic level marine ecosystem models (e.g. Tittensor et al. 2021). This integrative approach represents a step towards understanding ecosystem changes comprehensively.

##### Impacts: Implications for Dimethylsulfide

Abrupt shifts in phytoplankton productivity and diversity carry significant implications for the phytoplankton-aerosol-cloud feedback mediated by dimethylsulfide (DMS), as proposed by Charlson et al. (1987). DMS, a marine biogenic source of sulphur-containing aerosols such as sulphate, plays a key role in influencing the radiative properties of the atmosphere. For instance, a projected 24% reduction in the global sea-to-air DMS flux, potentially resulting from substantial changes in phytoplankton productivity and diversity, is estimated to warm the atmosphere by up to 0.76 K globally (Six et al. 2013). In the ocean, DMS originates from dimethylsulfoniopropionate (DMSP), an organosulphur compound primarily produced by phytoplankton. The intracellular ratio of DMSP to carbon biomass varies significantly among phytoplankton functional types, ranging up to four orders of magnitude (Stefels et al. 2007), highlighting the importance of phytoplankton diversity in DMS production. The production of DMSP and DMS will change as a result of not only warming but also acidification (Schwinger et al. 2017; Six et al. 2013). However, estimates on projected DMS changes using the current-generation climate models neither fully account for these environmental effects nor these models incorporate sufficient phytoplankton diversity (Bock et al. 2021).

**Fig. 6:**
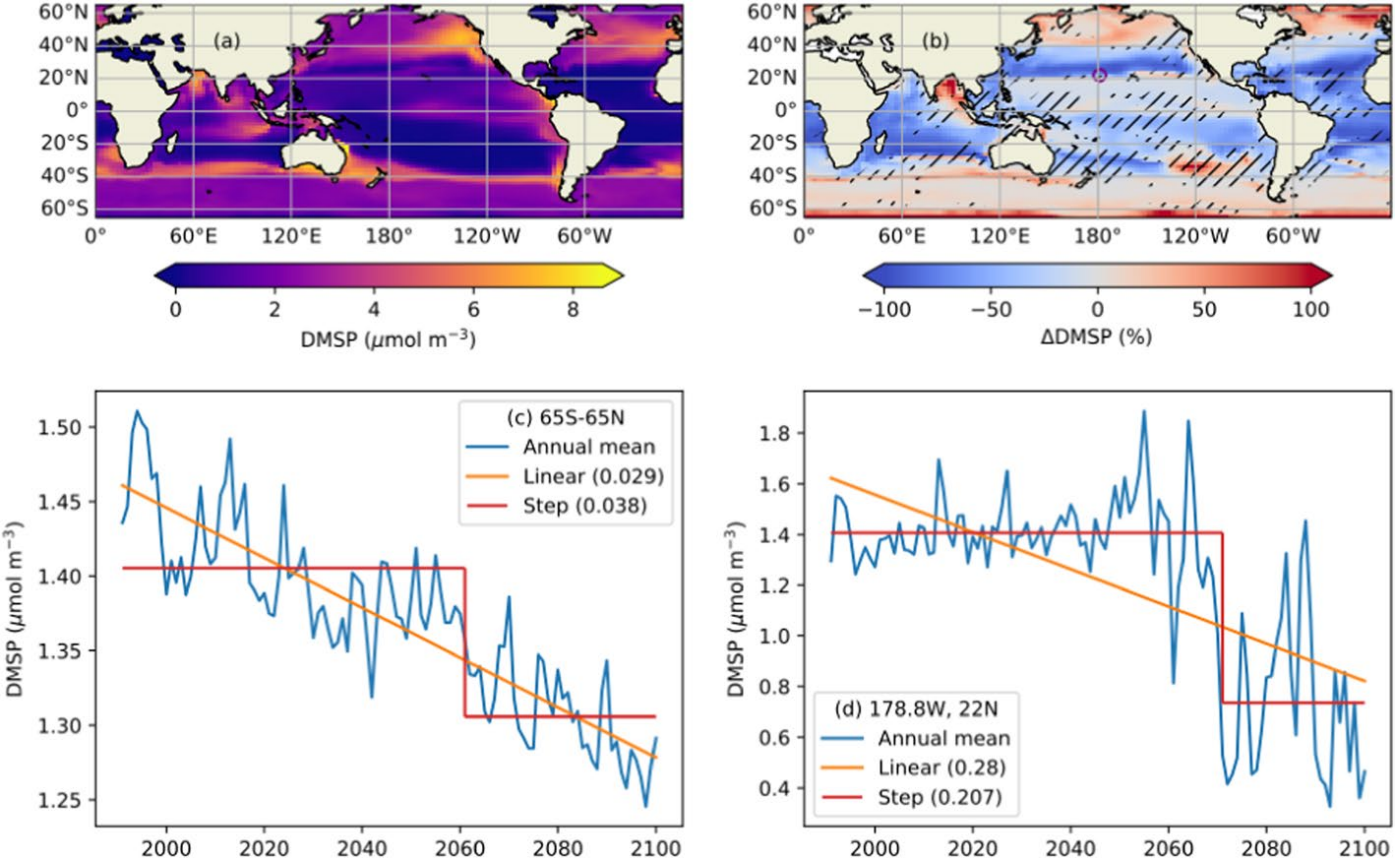
Abrupt shifts in dimethylsulfoniopropionate (DMSP) in the global ocean during the twenty-first century derived from the output of a 35-compartment phytoplankton model projection based on a high greenhouse gas emission scenario. **a** Global distribution of annual mean sea surface DMSP averaged over 2005–2024, **b** projected percentage change in annual mean sea surface DMSP averaged over 2081–2100 relative to the period 2005–2024, and time series of annual mean sea surface DMSP **c** averaged over 65°S-65°N and **d** at a location in the North Pacific subtropical gyre denoted by a purple circle in **b**. DMSP is derived by multiplying the modelled carbon biomass of dinoflagellates by 22, coccolithophores by 11, diatoms by 0.86, and diazotrophs/prokaryotes/picokaryotes by 0.0015, which are the observed mean intracellular ratios for these phytoplankton functional types (Stefels et al. 2007; M. Galí and J. Stefels, personal communication, September 2023). In **b**, hatches represent areas where the step-function outperforms the linear regression in fitting the projected time series over 1991–2100 at each grid point. In **c** and **d**, orange and red lines represent the best fit based on linear regression and step function, respectively. Numbers in parentheses represent the root mean square error of each fit. See the main text for details

Using the output of the 35-compartment phytoplankton model projection (Cael et al. 2021; Henson et al. 2021), we illustrate potential regional-scale abrupt shifts in DMSP during the twenty-first century. By multiplying the modelled phytoplankton biomass of the six functional types by their respective observed intracellular ratios, we derive the annual mean sea surface DMSP distribution averaged over the present period (2005–2024; Fig. 6a) and the projected percentage changes by the end of the twenty-first century (2081–2100; Fig. 6b). The DMSP time series from 1991 to 2100 at each grid point are fitted using both linear regression (Virtanen et al. 2020) and a step function through an offline change point detection method (Truong et al. 2020). The identification of abrupt changes is based on a comparison of the root mean square error (RMSE) of the step function to that of the linear fit, considering changes as abrupt when the ratio of the step-function RMSE to the linear-fit RMSE is less than 0.99.

Projected DMSP changes are characterised by a high (close to 100%) increase in sub-polar regions (beyond 40°N/S) and a decrease in the tropics and sub-tropics (Fig. 6b). Henson’s et al., (2021) work analyses the association with projected changes in phytoplankton biomass and notice that in the areas above DMS and phytoplankton biomass patterns are similar. However, there are regions, such as the Bay of Bengal and the Iceland Basin, where DMSP changes are prominent despite subtle biomass and diversity changes (Fig. 1a, b of Henson et al. 2021). Regression analysis indicates gradual DMSP increases in these regions (Fig. 6b). Abrupt shifts are projected to occur in various regions without a clear pattern regarding potential drivers, such as phytoplankton biomass and community composition.

In summary, our results suggest that the contribution of marine ecosystems to radiative forcing through DMS emission is likely to change gradually at the global scale (Fig. 6c), while abrupt changes may occur at the regional scale during the twenty-first century (Fig. 6b, d). These regional abrupt changes in DMS may be crucial for new particle formation in areas with low background cloud condensation nuclei, such as the Arctic (Sharma et al. 2012). It is crucial to note that our results are based on a basic parameterisation of intracellular DMSP-to-carbon ratios, intended to demonstrate the possibility of abrupt changes in DMSP in the coming decades. In reality, these ratios vary significantly within and among species, strongly depending on environmental conditions such as nutrient stress and acidification (Six et al. 2013). A more accurate projection requires a sophisticated parameterisation (McParland and Levine 2019). Further advancements in both modelling and remote sensing of phytoplankton functional types are essential for accurate quantification of DMSP in the ocean and monitoring the tipping point for the regional phytoplankton-aerosol-cloud feedback.

#### EO Needs and Opportunities

Detection and early warning of tipping points for these elements require further investigation into the various environmental factors influencing productivity and diversity, along with assessment through modelling. Long-term monitoring of phytoplankton productivity and diversity through remote sensing has proven effective in understanding global-scale patterns (Groom et al. 2019) and their responses to gradual climate changes (Cael et al. 2023). Continuous investment in this monitoring effort is essential to advance knowledge in the coming decades. Two ongoing satellite missions, the Global Change Observation Mission – Climate "SHIKISAI" (GCOM-C; https://global.jaxa.jp/projects/sat/gcom_c/) and the Plankton, Aerosol, Cloud, and ocean Ecosystem (PACE; https://pace.gsfc.nasa.gov/), offer prospects over the next decade, and additional missions are crucial for the future. Simultaneously, it is important to enhance the calibration of multiple missions to detect decadal changes.

Further improvements are needed in algorithms for identifying multiple phytoplankton groups, and these enhancements should occur concurrently with the parallel development of models. For instance, considering the significance of phytoplankton species composition in shaping the DMSP distribution, a top priority should be to increase the number of phytoplankton functional types in Earth System Models (ESMs), ideally to six as recommended by Stefels et al. (2007). Such an implementation would enable the assessment of the suitability of existing models with simpler complexity for projection studies (Bock et al. 2021) and open up new opportunities for further exploration.

### Zooplankton

#### Nature of Tipping Point

Marine zooplankton can be considered a tipping element in marine ecosystems due to their central role in maintaining the balance and functionality of oceanic food webs and biogeochemical cycles: marine zooplankton are in fact fundamental to ocean ecosystems and global biogeochemical cycles, serving as a critical link between primary producers, like phytoplankton, and higher trophic levels, including fish, seabirds and marine mammals. Through their feeding activities, zooplankton help regulate phytoplankton populations, thus influencing primary production and the overall energy flow within marine food webs. Additionally, zooplankton play a key role in the "biological pump," a process that contributes to carbon sequestration. By consuming phytoplankton and converting this organic matter into faecal pellets, which sink to the ocean floor, zooplankton facilitate the transport of carbon from the surface to the deep ocean, effectively removing it from the atmosphere and helping mitigate climate change (Steinberg and Landry 2017; Turner 2015). Tipping points and abrupt shifts in marine zooplankton populations can therefore be extremely important because they can trigger significant and potentially irreversible changes in marine ecosystems and global biogeochemical cycles.

Abrupt community shifts (ACSs) in zooplankton have been documented in various marine ecosystems, providing valuable insights into ecological dynamics. Notable instances include occurrences in the North Pacific during 1976/1977 and 1989, as extensively studied by Ebbesmeyer et al. (1991), Francis and Hare (1994), Hare and Mantua (2000), Polovina (2005), and Field et al. (2006). Specifically, the north-east Pacific witnessed ACSs in 1976/1977 (McGowan et al. 2003; Miller et al. 2004) and 1998/1999 (Peterson and Schwing 2003; Lavaniegos and Ohman 2007), while the north-west Pacific experienced shifts in 1976/1977, 1988/1989, and 1998 (Chiba et al. 2006; Kasai and Ono 2007; Sakurai 2007). The Humboldt Current in the southern Pacific also observed ACSs in 1968/1970 and 1984/1986 (Alheit and Niquen 2004; Alheit 2009). In the north-west Atlantic, a notable event occurred in 1989/1990, documented by Frank et al. (2005), Pershing et al. (2005), Greene and Pershing (2007), Greene et al. (2012), and Greene et al. (2013).

Additionally, abrupt community shifts have been identified in European seas during the late 1980s, affecting the North Sea (Reid et al. 2001; Weijerman et al. 2005); the Baltic Sea (Alheit et al. 2005; Moellmann et al. 2009), and the north-west European shelf seas (1987/1990) (Beaugrand 2004, Heath and Beare 2008; the Black Sea (Daskalov et al. 2007, Oguz 2007) and the western and eastern (Adriatic) Mediterranean Sea (Conversi et al. 2010). This synchronicity of these shifts was investigated by Conversi et al 2010 and Beaugrand et al 2015, among others.

In the late 1990s, ACSs were observed in the north-east Atlantic and adjacent seas, the Black Sea, and San Francisco Bay (Oguz et al. 2003; Cloern et al. 2010; Luczak et al. 2011; Beaugrand et al. 2013). These shifts coincided with a global temperature shift around the same time (Reid and Beaugrand 2012). The ACS in the late 1970s originated from changes in large-scale boreal winter circulation patterns over the North Pacific in the mid-1970s, altering the environmental regime of these oceanic regions (Miller et al. 1994; Tsonis et al. 2007).

**Fig. 7:**
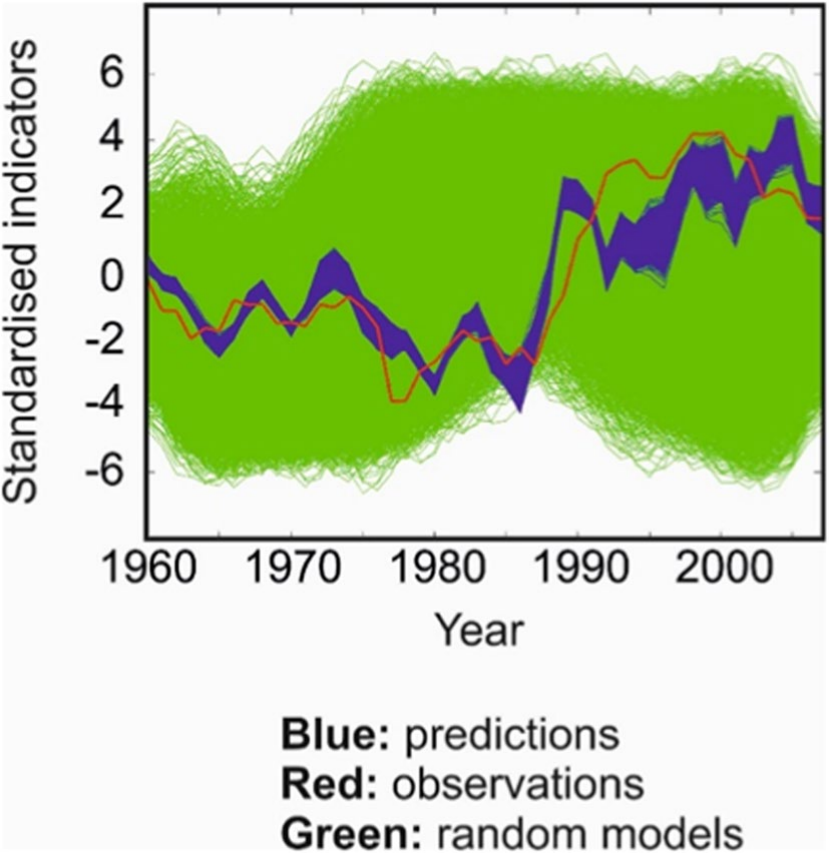
Comparison of predicted (blue) and observed (red) biological shifts. Predictions were made using a model based on the MacroEcological Theory on the Arrangement of Life (METAL). Observations were synthesised using a principal component analysis combining 14 oceanic regions (Atlantic, Southern and Pacific oceans and their adjacent seas). Green curves show results using random models. The y-axis represents arbitrary standardised indicators of the biological state of the ecosystems. Negative and positive states have contrasting effects on the ecosystems

Explanations for ACSs in zooplankton often reference the theory of alternative stable states (Holling 1973; Scheffer and Carpenter 2003; Scheffer 2009). While some ecosystem shifts may result from multiple attractors, establishing their existence remains challenging (Seekell et al. 2013). Processes and alternative stable states leading to ACSs have been recognised in lakes and some marine ecosystems (Nystrom et al. 2000; Scheffer and van Nes 2004; Scheffer 2009). Utilising the METAL theory (MacroEcological Theory on the Arrangement of Life, Beaugrand and Kirby 2018; Beaugrand 2023), Beaugrand (2015) demonstrated that many ACSs in zooplankton could arise from the nonlinear interaction between the ecological niche of individual species and environmental changes without necessarily invoking the existence of a tipping point. Subsequently, Beaugrand et al. (2019) developed a global numerical model based on METAL to understand and predict long-term biological changes in oceans, including ACSs, effectively reconstructing well-observed shifts in the pelagic ocean (Fig. 7).

**Fig. 8:**
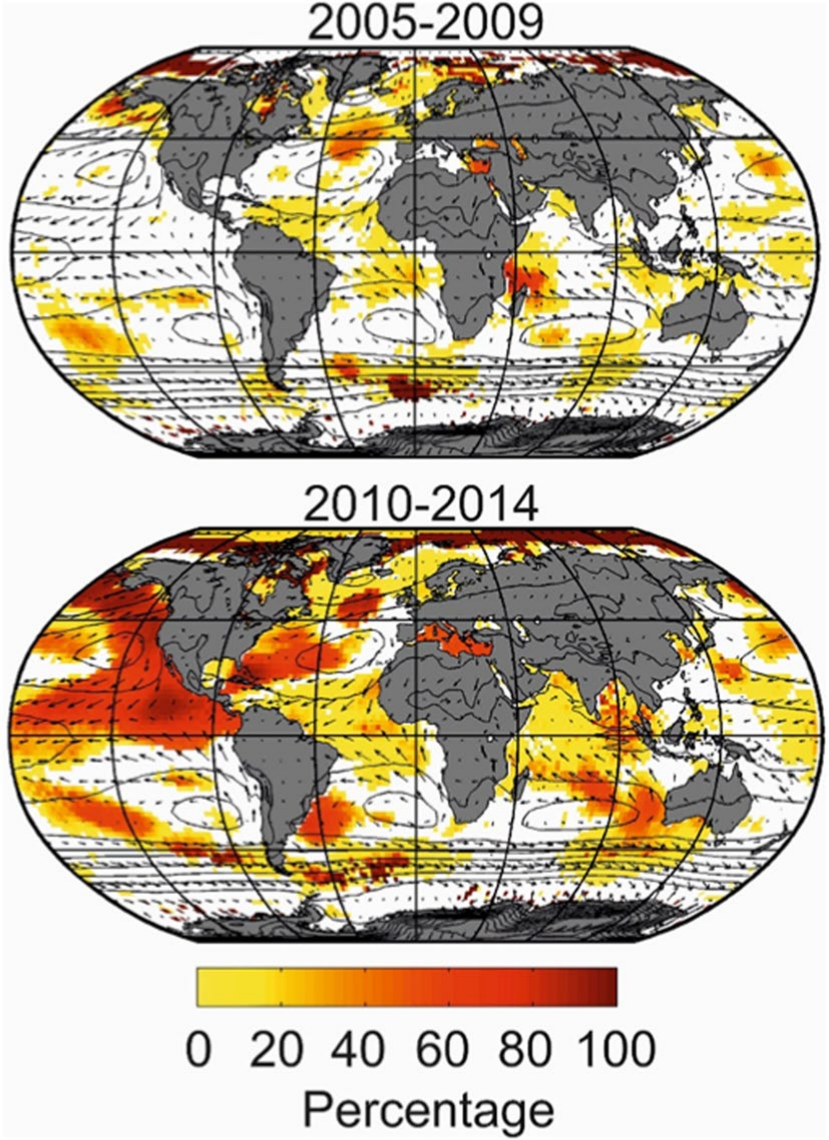
Results from the global METAL model for time periods 2005–2009 (top) and 2010–2014 (bottom). The red colours denote substantial biological shifts and the yellow colours indicate minor changes. No colour denotes an absence of biological shifts

The framework was subsequently applied globally for the period 1960–2015 to pinpoint areas where major biological shifts are likely. The analyses revealed that significant biological shifts may occur annually but are confined to a limited part of the ocean, averaging approximately 2.8% of the total oceanic area, translating to an average area of around 10 million km^2^ per year. Certain periods, such as 2005–2009, exhibited geographically restricted shifts, while others, like 2010–2014, demonstrated more extensive ones (see Fig. 8). The spatial extent of the biological shifts in 2010–2014 was particularly noteworthy and stemmed from the simultaneous occurrence of four phenomena: (i) a pronounced El Niño event (2015–2016),^1^ (ii) the Pacific warm blob (an anomaly featuring positive temperatures in the north-eastern part of the North Pacific), (iii) the Atlantic cold blob (a cold temperature anomaly in the central part of the North Atlantic), and (iv) strong positive temperature anomalies in the Arctic Ocean (Beaugrand et al. 2019).

#### EO Needs and Opportunities

Given the crucial role that marine zooplankton plays in ocean ecosystems and global biogeochemical cycles, there is a growing need to monitor zooplankton populations on large spatial and temporal scales. Earth Observation (EO) technologies offer potential solutions, though studying zooplankton from space presents unique challenges.

Direct observation of individual zooplankton from space is not feasible due to their small size. However, proxy measurements and indirect methods, such as using ocean colour data, show promise. For instance, zooplankton abundance can be inferred from satellite-derived chlorophyll concentrations, their primary food source. Strömberg et al. (2009; https://doi.org/10.1016/j.jmarsys.2009.02.004) demonstrated a relationship between chlorophyll-a concentrations and global zooplankton patterns. However, Friedland et al. (2018; https://doi.org/10.1111/geb.12717) showed that these relationships vary significantly across regions and seasons, complicating the use of satellite data for accurate zooplankton estimates.

Isolating the optical signature of zooplankton from other particulate matter in the water remains a significant challenge. Developing algorithms to separate the zooplankton contribution to ocean colour signals is crucial for improving the accuracy of zooplankton studies using EO data. Frouin et al. (2019; https://doi.org/10.3389/feart.2019.00145) reviewed advances in atmospheric correction for ocean colour remote sensing, underscoring its importance for the accurate estimation of bio-optical properties related to plankton. Persistent cloud cover in certain regions further complicates this issue, necessitating methods to work with limited or intermittent data. Moreover, managing both random and systematic errors is crucial to ensure the accuracy and reliability of the data.

Most zooplankton inhabit subsurface layers not directly observable from satellites, requiring innovative methods to infer subsurface conditions from surface observations. Techniques developed by Sauzède et al. (2015; https://doi.org/10.5194/essd-7-261-2015) for estimating vertical chlorophyll profiles from satellite data could potentially be adapted for zooplankton studies. Additionally, high-frequency satellite observations, such as those from the Geostationary Ocean Color Imager (GOCI), can capture short-term variability in plankton communities (Li et al. 2022;b). Behrenfeld et al. (2019; https://doi.org/10.1038/s41586-019-1796-9) utilised satellite lidar to observe diel vertical migration of zooplankton, though this technology is not yet widely available.

Distinguishing between different zooplankton species or functional groups from space is extremely challenging. Alvain et al. (2008; https://doi.org/10.1029/2007GB003154) created a method to identify phytoplankton functional types from ocean colour data, which could serve as a model for similar approaches in zooplankton research.

Another challenge is the validation of zooplankton-related products derived from satellite data. Extensive in-situ sampling is necessary (Druon et al., 2019; https://doi.org/10.1038/s41598-019-41212-2). However, combining satellite data with in-situ measurements involves substantial technical and methodological challenges due to differences in scale, coverage, and measurement techniques.

The examination of abrupt shifts and their attribution to environmental drivers, detailed in Sect. 3.2.1, highlights the critical significance of global-scale coverage encompassing both physical parameters like sea surface temperature (SST) and biological variables such as colour, facilitated by EO. Coordinated international efforts are essential for advancing high-resolution remote sensing and data integration capabilities to handle the large volumes of high-resolution data required to accurately represent the Earth's surface. Addressing inter-sensor biases is also critical for long-term monitoring and avoiding false trends (Balsamo et al., 2019; https://doi.org/10.3390/rs11080941; Sathyendranath et al., 2023; https://doi.org/10.1007/s10712-023-09805-9).

### Higher Trophic Levels and Fisheries

#### Nature of Tipping Point

Pelagic ecosystems, particularly over continental shelves, do not operate in isolation, and the nonlinear interaction between plankton species and environmental changes can have cascading effects throughout the entire trophic food web. Numerous instances in the scientific literature (Lindley et al. 2010; Luczak et al. 2011, 2012) exemplify this phenomenon. For instance, a study revealed a surge in the population of balearic shearwater (*Puffinus mauretanicus*) during the mid-1990s in the north-east Atlantic (17°W-4°E and 48°N-60°N). Concurrently, significant changes in a zooplankton composition index from the Continuous Plankton Recorder (CPR) survey were noted, along with a stepwise increase in the abundance of sardine and anchovy, the primary diet of the seabird (Luczak et al. 2011).

In regions over continental shelves, pelagic and benthic ecosystems are intricately linked, and alterations in pelagic ecosystems have pronounced impacts on benthic ecosystems (Lindley et al. 2010; Luczak et al. 2012). Profound transformations in the North Sea ecosystems, occurring at the end of the 1980s and from the middle to the end of the 1990s, were observed (Reid et al. 2001; Beaugrand et al. 2014). The abrupt rise in temperature significantly disrupted the trophodynamics of pelagic ecosystems, subsequently affecting benthic communities (Lindley et al. 2010). In the late 1990s, an increase in swimming crabs further influenced the reproductive success of lesser black-backed gull (*Larus fuscus graelsii*), establishing a connection between marine ecosystem shifts and terrestrial ecology (Luczak et al. 2012).

Alterations in the composition of North Sea zooplankton also had a considerable impact on commercially exploited fish, such as Atlantic cod (*Gadus morhua*). Post the late 1980s, changes in small planktonic crustaceans, crucial as the primary food for larval cod, occurred with a reduction in size, abundance, and biomass (Beaugrand et al. 2003). The timing of the main peak in copepod abundance shifted later in the year, coinciding with the period when fish larvae had matured and were no longer reliant on these small prey organisms. Combined with overfishing, these abrupt shifts adversely affected larval cod recruitment.

A novel model based on the METAL theory has demonstrated how overfishing and environmental changes can interact to impact cod recruitment and biomass in the North Sea (Beaugrand et al. 2022). The identification of a tipping point and the suggestion of hysteresis under certain conditions emphasis e the complex dynamics. Overexploitation can swiftly deplete fish stocks (Christensen et al. 2003; Pauly et al. 2003). Beaugrand et al. (2022) illustrated that, in favourable environmental conditions and with a hypothetical moratorium, recovery may occur, but the time required is lengthier than the duration of fishing-induced depletion. The recovery duration is influenced by the interaction between the low stock level and the initially slow exponential population growth phase, dependent on the stock's growth coefficient. However, when environmental conditions become unfavourable, recovery may become exceedingly challenging, spanning decades or even be impossible if adverse conditions persist, indicating irreversibility.

This example also elucidates why, despite a fishing moratorium near Newfoundland, recovery, albeit partial, took more than two decades (DFO 2018). Notably, recovery proves problematic for numerous other fish stocks (e.g. haddock, flatfish, Hutchings 2000). Hence, preventing collapse emerges as a more attainable goal than attempting to reverse it. Rebuilding a stock remains achievable only if environmental conditions remain conducive (Möllmann et al. 2021).

#### EO Needs and Opportunities

In this section, we review the significant challenges and promising opportunities associated with using Earth Observation (EO) to study higher trophic levels and fisheries.

Challenges:

1. Indirect Observations: One major obstacle is the inability to directly observe higher trophic species from space, which necessitates the use of proxy measurements and habitat modelling. This indirect approach is complicated by the complex, nonlinear relationships between environmental conditions and higher trophic levels, as well as time lags between observable environmental changes and their effects on species. These factors make it difficult to establish clear cause-and-effect relationships and accurately interpret EO data (Grémillet and Boulinier, 2009; https://doi.org/10.3354/meps08212). The temporal mismatch between environmental changes and biological responses can obscure true relationships between oceanographic conditions and species distributions or abundances (Visser and Both, 2005; https://doi.org/10.1098/rspb.2005.3356). This issue is particularly pronounced for long-lived species, where environmental effects may only become apparent after significant delays (Durant et al., 2007; https://doi.org/:10.3354/cr033271). Additionally, the limited historical record of EO data (Swingedouw et al. 2020) and methodological consistency problems when reconstructing long time series from different sensors (Sathyendranath et al., 2023; https://doi.org/10.1007/s10712-023-09805-9) further complicate the analysis.
2. Data integration Issues: Similar to the other biological components, Integrating EO data with in-situ observations, catch data, and ecosystem models is crucial for a comprehensive understanding of marine ecosystems but faces several challenges. Differences in data resolution, scale, and format can hinder this integration (Rose et al., 2015; https://doi.org/10.1111/cobi.12397). The processes affecting fisheries and higher trophic levels often occur at scales that do not align with the resolution of available EO data, potentially missing fine-scale movements and leading to challenges in representing ecosystem dynamics (Rose et al., 2015; https://doi.org/10.1111/cobi.12397; Kavanaugh et al., 2016; https://doi.org/10.1093/icesjms/fsw086).

Opportunities:

1. Monitoring Fishing Activities: EO has proven valuable in monitoring fishing activities. Kroodsma et al. (2018; https://doi.org/10.1126/science.aao5646) utilised AIS data and satellite imagery to create a global map of fishing effort, providing insights into the spatial and temporal patterns of fishing activities. This information is essential for enforcing fishing regulations and designing effective marine protected areas.
2. Habitat Modelling: Significant progress has been made in using satellite-derived oceanographic data to model and map potential habitats for species such as the blue shark (Druon et al., 2022; https://doi.org/10.3389/fmars.2022.828412). Muller-Karger et al. (2018; https://doi.org/10.3389/fmars.2018.00211) proposed a framework for integrating satellite observations with in-situ data and models to support ecosystem-based management of fisheries and marine resources. However, these approaches still require refinement.
3. Data Integration Advances: A strategic combination of remote sensing, in situ observations, and appropriate modelling is essential (Stuart et al. 2011). EO technology addresses critical gaps, providing an opportunity for all countries and individuals, even those without the capacity to collect essential data, to contribute to local solutions for global challenges (e.g. Almar et al. 2022). Recent advances in data assimilation techniques have shown promise in bridging the gap between satellite observations and ecosystem models (de Souza et al., 2016; https://doi.org/10.1371/journal.pone.0163760; Schwartz-Belkin & Portman, 2023; https://doi.org/10.1016/j.ocecoaman.2022.106280). By implementing models trained on a combination of EO and in situ data, Hazen et al. (2018) introduce an innovative multispecies and dynamic approach that utilises daily satellite data to monitor ocean features, aligning the scales of management, species movement, and fisheries.
4. Policy advances: Policy makers must understand the significance of satellite EO data in evidence-based decision-making. For example, NASA's Applied Sciences Programme within the Earth Science Division offers free training on the practical application of satellite data, covering objectives such as fisheries management (Andries et al. 2022). Global Fishing Watch builds geospatial datasets and hosts an online mapping platform that allows anyone with internet access to monitor various types of ocean-going vessels, sharing data and map products with scientists and practitioners, notwithstanding challenges discussed in (Drakopulos et al. 2023).

Future efforts should focus on improving our ability to detect and predict tipping points in marine ecosystems. This can be achieved through higher resolution satellite data, improved algorithms for detecting subtle changes in ecosystem indicators, and better methods for integrating multiple data sources to identify early warning signs of ecological shifts.

Integrating remote sensing EO technology with environmental decision-making enhances the efficiency and sustainability of solutions. Therefore, the broader scientific community must consistently support programs that enable the application of satellite EO data for global societal benefit and the stewardship of our planet (Kansakar & Hossain 2016).

### Marine Biodiversity and Biomass

#### Nature of Tipping Point

While human activities, such as fishing, pollution, and species invasion, exert a significant impact on pelagic marine biodiversity, global climate change also plays a substantial role. However, assessing the extent of this stressor and the presence of tipping points remains challenging due to data and theoretical approach limitations.

A theoretical framework based on METAL estimated the vulnerability of global ocean biodiversity to climate change (Beaugrand et al. 2015). This framework suggested that climate change could swiftly reorganise marine biodiversity across extensive oceanic regions. Observations have since confirmed an increase in marine biodiversity in extratropical regions Hiddink and Hofstedeter 2008; Gordó-Vilaseca et al. 2023), aligning with the model's predictions. Rapid and substantial changes in marine biodiversity occurred in the north-east Atlantic and its adjacent seas in the late 1980s, influencing the entire trophodynamics of the North Sea across phytoplankton, fish, pelagic and benthic realms, and extending to terrestrial ecosystems (Beaugrand 2009). Although no clear tipping point has been identified, these changes are believed to result from the nonlinear interaction between species' ecological niches and shifts in the environmental regime (Beaugrand and Kirby 2018).

Comparisons of biodiversity changes expected by the end of this century with those observed between the last glacial maximum (LGM; about 20,000 years ago) and today, or between the mid-Pliocene (2.3 million years ago) and today, offer insights. The study highlights that the intensity of biodiversity reorganisation depends on the magnitude of warming (Beaugrand et al. 2015). Under scenarios of small global warming (RCP2.6), biological changes would be relatively benign, reflecting a modest percentage of historical changes. However, for moderate warming (RCP4.5), changes in marine biodiversity are considerably more extensive and robust than those observed over the past 50 years. In the case of severe global warming (RCP6.0 and 8.5), a substantial portion of the global ocean experiences a change in marine biodiversity equivalent to or exceeding that observed between the LGM/mid-Pliocene and today. This underscores the significant impact of climate warming on marine biodiversity. Any reorganisation of marine biodiversity is bound to influence species interactions and, consequently, ecosystem functioning, provisioning, and regulating services (Cheung et al. 2009), emphasising the importance of understanding the effects of climate change on biodiversity.

#### EO Needs and Opportunities

Earth Observation (EO) is crucial for species distribution mapping, particularly through habitat mapping. Satellites use spectral bands to identify marine habitats like coral reefs, seagrass beds, and kelp forests, with changes in these habitats indicating shifts in dependent species. For example, Landsat satellites have been instrumental in mapping coral reefs globally. (Li et al. 2021) used Landsat to create a global coral reef map, essential for understanding coral-dependent species distributions and detecting ecosystem tipping points. EO data combined with species distribution models helps predict suitable habitats and guide conservation efforts, as demonstrated by Yati et al. (2024; https://doi.org/10.1016/j.marenvres.2024.106540), who used this approach to propose marine protected areas. Monitoring changes in predicted habitats over time allows detection of species distribution shifts, potentially indicating broader ecosystem changes.

EO also facilitates direct observation of larger marine species or large multi-individual aggregations. High-resolution satellite imagery, such as WorldView-3, has been used to detect and count whales, as shown by Cubaynes et al. (2019; https://doi.org/10.1111/mms.12544). Similarly, satellites equipped with ocean colour sensors, such as MODIS, can map harmful algal blooms, which impact marine ecosystems, as demonstrated in studies by Zhao et al. (2020; https://doi.org/10.1016/j.envadv.2020.100008) and others.

Satellite imagery is also useful in mapping seabird colonies, such as Lynch et al.'s (2012; https://doi.org/10.1007/s00300-011-1138-3) survey of Adélie penguins in Antarctica.

**Fig. 9:**
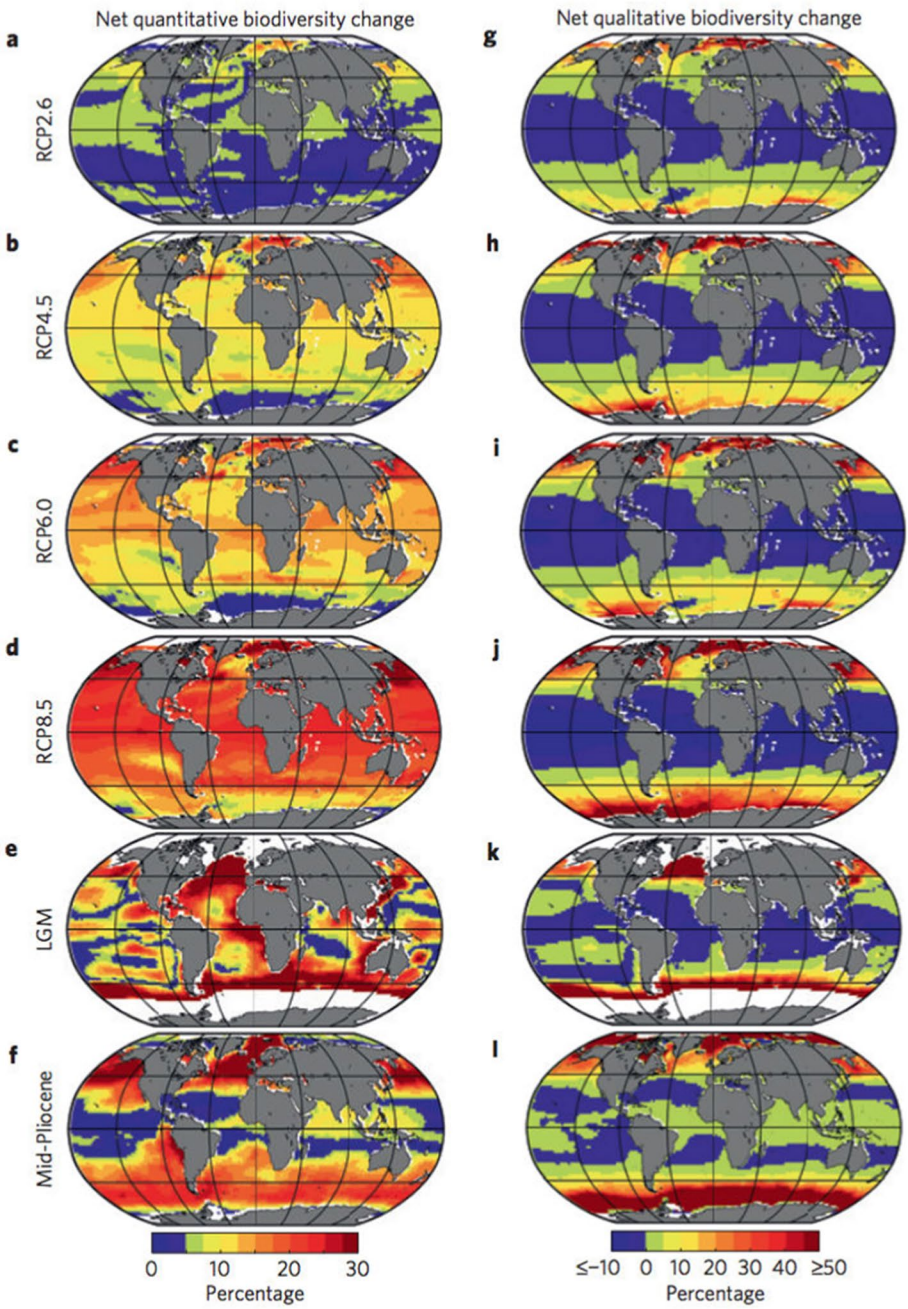
Past and future quantitative (left-hand maps) and qualitative (right-hand maps) change in marine biodiversity. Maps on top four rows **a**–**d** and **g**–**j** show the difference between projected future (2081–2100) and present (2006–13) under the four emissions scenarios. Maps on bottom two rows show difference between the last glacial maximum (LGM, maps **e** and **k**) and 1960–69, and the Mid-Pliocene (maps **f** and **l**) and 2006–13. Regions of largest percentage change shown as oranges and reds, and smaller changes as blues and greens. From Beaugrand and colleagues (Beaugrand et al. 2015)

Evaluating and monitoring future biodiversity requires robust models, similar to those used in generating Fig. 9. These models should be calibrated and evaluated based on recent biodiversity changes, using environmental parameters often derived from remote sensing.Additionally, empirical and data-driven early warning approaches, as demonstrated by studies like Dakos et al. (2010) and Verbesselt et al. (2016), present promising avenues. These approaches can be directly fuelled by Earth Observation (EO) data or utilise the outputs of ecosystem models.

Future EO needs and opportunities include:

- Hyperspectral sensors for improved habitat discrimination
- Higher temporal resolution data from geostationary satellites
- Improved atmospheric correction algorithms for coastal waters
- LiDAR technology for detailed coastal topography and bathymetry
- Advanced SAR systems for all-weather monitoring of marine environments

The development of data fusion techniques and integrated satellite platforms, which combine data from optical, radar, and thermal sensors, holds significant promise, e.g. Moroni et al. (2016; https://doi.org/10.1016/j.marpolbul.2015.07.045). This multi-sensor approach, coupled with advanced algorithms and AI, as shown by Guirado et al. (2019; https://doi.org/10.1038/s41598-019-50795-9) for detecting whales, is revolutionising ecological research.

Advancements in EO technology, like higher resolution sensors optimised for coastal and inland waters, offer new opportunities for monitoring complex environments. Kutser et al. (2016; https://doi.org/10.1080/01431161.2016.1186852) reviewed these methods, emphasising their importance for fine-scale ecological monitoring. Developing new biodiversity metrics based on EO data is crucial for conservation, as discussed by Pettorelli et al. (2016; https://doi.org/10.1002/rse2.15), while long-term data consistency, as stressed by Mélin et al. (2017; https://doi.org/10.1016/j.rse.2017.03.039), is essential for tracking ecological changes.

Combining EO with in-situ data enhances the accuracy of biodiversity assessments. Muller-Karger et al. (2018; https://doi.org/10.3389/fmars.2018.00211) proposed a framework for such integration, which is vital for a holistic understanding of ecosystems. Advances in satellite-derived bathymetry and seafloor classification, as reviewed by Hedley et al. (2016; https://doi.org/10.3390/rs8020118), hold promise for better mapping of underwater habitats. Integrating satellite observations with emerging techniques like environmental DNA (eDNA) sampling, or data from biogeochemical-Argo floats could provide a more comprehensive picture of marine biodiversity, as explored by Yamasaki et al. (2017; https://doi.org/10.1016/j.cosust.2018.03.005) and Sauzède et al. (2016; https://doi.org/10.1002/2015JC011408).

Cloud-based platforms and standardised data products are key for timely biodiversity assessments. Duffy et al. (2019; https://doi.org/10.3389/fmars.2019.00317) emphasised the importance of near-real-time EO data for conservation.

Looking ahead, satellite-based acoustic sensors could monitor underwater noise levels, potentially affecting marine life. While still in its early stages, this innovative approach, discussed by Sun et al. (2021; https://doi.org/10.3390/s21237849), represents the kind of innovative thinking needed to fully leverage EO for marine biodiversity monitoring. These advancements will significantly enhance our ability to monitor and understand marine ecosystems using Earth Observation technologies.

## Synthesis and Prospects

Specific needs for Earth Observation (EO) depend strongly on the detail of the Tipping Element under consideration, and the reader is referred to the individual TE discussions in Sects. 2 and 3 of this paper. However, some common themes emerge. These themes are not all unique to ocean tipping points, but the nature of the ocean and of abrupt changes places particular emphasis on some of them. References are given beside each point below to relevant TE-specific discussion in earlier sections:

- The value of long-term, uninterrupted and homogeneous timeseries is clear in several areas, and is particularly important when studying abrupt changes that span decadal timescales (e.g. AMOC §2.2.2, ecosystem dynamics, especially involving long-lived species, §3.2.2, 3.3.2). Issues of continuity and cross-calibration should therefore be high on the agenda of future mission planning, as well as resolving apparent differences between alternative instruments or products (e.g. polar SSS, §2.2.2)
- An important role for EO emerges in monitoring and early detection of changes in critical fine scale processes (e.g. transports through Arctic gateways §2.3.2, 2.4.2, submesoscale dynamics §2.5.2, patchy and moving planktonic systems §3.1.2, 3.2.2). In many cases the value of EO for this purpose can only be realised through combination with in situ, and model data (especially for processes involving the sub-surface ocean, which is not directly accessible to EO). Machine Learning, perhaps trained on high-resolution process-resolving models, provides particular new opportunities to integrate multiple datasets, augmenting the spatial and temporal resolution, and vertical coverage, of EO.
- Another important role for EO is in providing ‘truth’ data to assess and develop parametrisations of fine scale processes for use in coarser models (e.g. air-sea-ice interactions §2.2.2, 2.3.2). Many models used for large-scale climate assessments lack resolution in key areas (e.g. in space, time or ecosystem complexity).
- In other cases, state-of-the-art models (especially regional or subsystem-specific models) are ahead of current EO capabilities in resolution or complexity (e.g. multi-species ecosystem models, §3.3, 3.4). This creates a demand for higher resolution or accuracy than is possible with current EO systems. As well as driving future technological improvements, the existence of complex models provides opportunities to develop improved algorithms to maximise the information extracted from existing instruments, including EO-derived proxies for variables that cannot be observed directly. Again, Machine Learning is opening up new possibilities in this area.
- Combination or joint analysis of several data types is challenging, and in some cases methods of consistently assimilating multiple data types into (inevitably biased) models are a limiting factor in obtaining full value from existing EO datasets (e.g. for decadal prediction systems, §2.2.2). Developments in modelling and multivariate data assimilation must therefore proceed in tandem with future observational developments if the full potential of EO is to be realised.
- While data assimilating models, incorporating multiple data streams, offer a long-term path to monitoring and early warning, alternative methods of analysis based directly on EO data can provide valuable insights. For example, empirical methods for identifying approaching tipping through linking the changing properties of timeseries to simple conceptual models of tipping dynamics (e.g. critical slowing down §2.1, or space-for-time, §2.2), are well suited to the high spatial or temporal resolution of many EO datasets. The advent of high-resolution models and machine learning techniques opens up many new possibilities for training and evaluating such methods.
- It will never be possible to observe everything we would like to in sufficient detail. So inevitably, indirect or proxy relationships will need to be used between observed variables and the actual system variable of interest (e.g. various AMOC proxies §2.1.2, key elements of the oxygen cycle §2.6.2, indicators of ecosystem structure §3.2.2, 3.3.2). When developing empirical early warning indicators and proxies, it will be important to build confidence through model studies that such methods can detect the changes that may occur in future, as well as what has happened in the past (e.g. for AMOC §2.1.1). For long timescale processes palaeoclimatic data/model studies may be an important source of calibration (e.g. biogeochemical processes §2.6). It will also be important to establish that relationships developed for one region are also applicable to other regions (e.g. for planktonic ecosystems §3.2.2) Specific attention should be paid to the combination of different observables, both EO and in situ, to increase the robustness of warning signals (e.g. a combination of temperature and salinity, both surface and subsurface and from different locations, to derive an early warning indicator for AMOC or SPG, §2.1.2, 2.2.2).
- The multivariate approaches outlined above imply an increasing need for improvements in data standardisation and accessibility, despite the challenges of differing space–time scales, timeliness, quality assurance and error characteristics (see e.g. discussion in §3.3.2). This presents a particular challenge to the international scientific community, as solutions developed for a particular problem may not be useful in the context of another.

As the focus of climate science moves from establishing the challenges of climate change to supporting societal responses to those challenges, so the demand for detail in monitoring changes in the climate system increases. The likelihood of crossing climate tipping points is expected to increase as the climate warms (IPCC 2021), and there is an urgent need for a joined-up approach, integrating EO and in situ observations with climate modelling, downstream impacts assessment and societal decision-making and policy (Wood et al. 2023). Earth Observation has a key role to play in avoiding the worst risks of climate tipping, and in building resilience to those risks that cannot be avoided.

## Abbreviations

ACSs: Abrupt community shifts
AI: Artificial intelligence
AIS: Automatic identification system
AMOC: Atlantic meridional overturning circulation
ASI: Infrared atmospheric sounding interferometer
bbp: Particulate back scattering
BG: Beaufort gyre
BH: Beaufort high
CH: Cold halocline
CIMR: Copernicus imaging microwave radiometer
CI: Climate change initiative
CMIP: Climate model intercomparison project
DMS: Dimethylsulfide
DMSP: Dimethylsulfoniopropionate
ENSO: El Niño Southern Oscillation
EO: Earth observation
ESM: Earth system models
EWIs: Early warning indicators
GCMs: General circulation models
GHGs: Greenhouse gases
HILL: High impact low likelihood
IL: Icelandic low
ISSI: International space science institute
ITCZ: Intertropical convergence zone
LGM: Last glacial maximum
METAL: MacroEcological theory on the arrangement of life
ML: Machine learning
NAHosMIP: North Atlantic Hosing model intercomparison project
NAO: North Atlantic Oscillation
nNLS: Near-shore non-large meander
OAEs: Oceanic anoxic events
ODZs: Oxygen deficient zones
oNLS: Off-shore non-large meander
POC: Particulate organic carbon
RFI: Radio frequency interference
RMSE: Root mean square error
Rrs: Remote sensing reflectances
SCICEX: Scientific ice expeditions
SPG: Atlantic sub-polar gyre
SMAP: Moisture active passive
SMOS: Soil moisture and ocean salinity
SROCC: Special report on the ocean and cryosphere in a changing climate
SSH: Sea surface height
SSS: Sea Surface salinity
SST: Sea surface temperature
TE: Tipping element
TipMIP: Tipping point modelling intercomparison project
tLS: Typical large meander
TP: Tipping point
TPD: Transpolar drift

## Footnotes

1 *Note that the calculation of a shift for a given year was based on a three-year period. Therefore, the time periods shown here embrace 1 year before and after in the calculation; 2005-2009 and 2010-2014 include 2004-2010 and 2009-2015.*
